# *Leishmania* Genome Dynamics during Environmental Adaptation Reveal Strain-Specific Differences in Gene Copy Number Variation, Karyotype Instability, and Telomeric Amplification

**DOI:** 10.1128/mBio.01399-18

**Published:** 2018-11-06

**Authors:** Giovanni Bussotti, Evi Gouzelou, Mariana Côrtes Boité, Ihcen Kherachi, Zoubir Harrat, Naouel Eddaikra, Jeremy C. Mottram, Maria Antoniou, Vasiliki Christodoulou, Aymen Bali, Fatma Z. Guerfali, Dhafer Laouini, Maowia Mukhtar, Franck Dumetz, Jean-Claude Dujardin, Despina Smirlis, Pierre Lechat, Pascale Pescher, Adil El Hamouchi, Meryem Lemrani, Carmen Chicharro, Ivonne Pamela Llanes-Acevedo, Laura Botana, Israel Cruz, Javier Moreno, Fakhri Jeddi, Karim Aoun, Aïda Bouratbine, Elisa Cupolillo, Gerald F. Späth

**Affiliations:** a Institut Pasteur—Bioinformatics and Biostatistics Hub—C3BI, USR 3756 IP CNRS, Paris, France; b Unité de Parasitologiemoléculaire et Signalisation, Institut Pasteur, Paris, France; c Laboratory on Leishmaniasis Research, Oswaldo Cruz Institute—Fiocruz, Rio de Janeiro, Brazil; d Laboratoire d’Eco-épidémiologieparasitaire et Génétique des Populations, Institut Pasteur d’Algérie, Algiers, Algéria; e Centre for Immunology and Infection, Department of Biology, University of York, York, United Kingdom; f Laboratory of Clinical Bacteriology, Parasitology, Zoonoses and Geographical Medicine, School of Medicine, University of Crete, VassilikaVouton, Heraklion, Greece; g Laboratory of Transmission, Control and Immunobiology of Infections (LTCII), Institut Pasteur de Tunis, Tunis-Belvédère, Tunisia; h Université Tunis El Manar, Tunis, Tunisia; i The Institute of Endemic Diseases, University of Khartoum, Khartoum, Sudan; j Molecular Parasitology Unit, Institute of Tropical Medicine, Antwerp, Belgium; k Department of Biomedical Sciences, University of Antwerp, Antwerp, Belgium; l Molecular Parasitology Laboratory, Microbiology Department, Hellenic Pasteur Institute, Athens, Greece; m Laboratory of Parasitology and Vector-Borne-Diseases, Institut Pasteur du Maroc, Casablanca, Morocco; n WHO Collaborating Centre for Leishmaniasis, Instituto de Salud Carlos III, Madrid, Spain; o Research Laboratory Medical Parasitology, Biotechnology and Biomolecules, Institut Pasteur de Tunis, Tunis-Belvédère, Tunisia; Yale University School of Public Health

**Keywords:** *Leishmania*, aneuploidy, evolution, gene copy number variation, genomic adaptation, telomeric amplification

## Abstract

Protozoan parasites of the genus Leishmania adapt to environmental change through chromosome and gene copy number variations. Only little is known about external or intrinsic factors that govern Leishmania genomic adaptation. Here, by conducting longitudinal genome analyses of 10 new Leishmania clinical isolates, we uncovered important differences in gene copy number among genetically highly related strains and revealed gain and loss of gene copies as potential drivers of long-term environmental adaptation in the field. In contrast, chromosome rather than gene amplification was associated with short-term environmental adaptation to *in vitro* culture. Karyotypic solutions were highly reproducible but unique for a given strain, suggesting that chromosome amplification is under positive selection and dependent on species- and strain-specific intrinsic factors. We revealed a progressive increase in read depth towards the chromosome ends for various Leishmania isolates, which may represent a nonclassical mechanism of telomere maintenance that can preserve integrity of chromosome ends during selection for fast *in vitro* growth. Together our data draw a complex picture of Leishmania genomic adaptation in the field and in culture, which is driven by a combination of intrinsic genetic factors that generate strain-specific phenotypic variations, which are under environmental selection and allow for fitness gain.

## Abstract (precis)

Protozoan parasites of the genus Leishmania cause severe human and veterinary diseases worldwide, termed leishmaniases. A hallmark of Leishmania biology is its capacity to adapt to a variety of unpredictable fluctuations inside its human host, notably pharmacological interventions, thus, causing drug resistance. Here we investigated mechanisms of environmental adaptation using a comparative genomics approach by sequencing 10 new clinical isolates of the L. donovani, L. major, and L. tropica complexes that were sampled across eight distinct geographical regions. Our data provide new evidence that parasites adapt to environmental change in the field and in culture through a combination of chromosome and gene amplification that likely causes phenotypic variation and drives parasite fitness gains in response to environmental constraints. This novel form of gene expression regulation through genomic change compensates for the absence of classical transcriptional control in these early-branching eukaryotes and opens new venues for biomarker discovery.

## INTRODUCTION

Protozoan parasites of the genus Leishmania are transmitted by female blood-feeding sand flies and can cause severe diseases in infected humans and animals. The success of this pathogen relies on its capacity to sense changes in various host environments that trigger a series of distinct developmental transitions (1). Inside phlebotomine insect vectors, noninfectious procyclic promastigote parasites differentiate into highly infectious metacyclic promastigotes, which are transmitted to vertebrate hosts during a blood meal, where they develop into the disease-causing amastigote form inside host macrophages (2, 3). Aside from stage differentiation, Leishmania parasites seem to adapt to a variety of environmental fluctuations encountered in their hosts, with important consequences for infection outcome, such as drug treatment. Phenotypic shifts in Leishmania have been linked to genome plasticity, with frequent copy number variations (CNVs) of individual genes or chromosomes linked to drug resistance (4–9) or tissue tropism (10, 11). A better insight into molecular and genetic mechanisms underlying Leishmania genetic diversity and evolution of new phenotypes is therefore essential to understand parasite pathogenicity and hence the epidemiology of Leishmania infection.

Combining DNA sequencing (DNA-seq) and transcriptome sequencing (RNA-seq) analyses of karyotypically distinct Leishmania donovani field isolates and experimental clones, we recently established a direct correlation between transcript abundance and chromosome amplification (12, 13)—a form of genomic regulation of gene expression levels that compensates for the absence of classical transcriptional control in these early-branching eukaryotes (10, 14, 15). Using the L. donovani LD1S experimental strain and conducting *in vitro* evolutionary experiments, we demonstrated the highly dynamic, reversible, and reproducible nature of parasite karyotypic changes and correlated chromosome amplification to fitness gains in culture (13). Using recent clinical isolates of L. donovani, we demonstrated that such karyotypic changes were strain specific (12), suggesting a potential link between the genetic background of the parasite and its karyotype plasticity (12, 16). Despite the potential relevance of genomic adaptation in shaping the parasite pathogenic potential, only little is known about the dynamics of gene and chromosome CNVs in Leishmania field isolates while they evolve to adapt to new environments. Here we address this important open question by comparing the genomes of 10 clinical isolates belonging to three different Leishmania complexes (L. donovani, L. major, and L. tropica) from eight geographical regions. Read depth analysis revealed gene and chromosome CNVs as potential drivers of long-term and short-term adaptation, respectively. Isolates during early and later stages of culture adaptation showed reproducible karyotypic changes for a given strain, providing strong evidence that chromosomal amplification is under positive selection. Significantly, these changes occurred in an individualized manner in even highly related strains, thus implicating for the first time environment-independent intrinsic genetic factors affecting Leishmania karyotypic adaptation.

## RESULTS

### Analyzing the evolutionary relationship among *Leishmania* strains.

Ten Leishmania strains belonging to the L. tropica, L. major, or L. donovani complexes were obtained from different sources and regions (see Materials and Methods and see Table S1 at GitLab [https://gitlab.pasteur.fr/gbussott/Leishmania_genome_dynamics_during_environmental_adaptation_reveals_strain_specific_differences/]), and parasites from early passage (passage 2) and later culture passages (passage 5 [designated EP and EP + 3, respectively]) were subjected to sequencing analysis (see Fig. S1 in the supplemental material and Table S2 at GitLab).

10.1128/mBio.01399-18.1 FIG S1 Overview of experimental design. Clinical isolates were obtained from infected patients or dogs, placed in culture under standardized conditions, and maintained for a defined number of passages *in vitro*. Promastigotes from logarithmic culture at passage 2 (early passage [EP]) or passage 5 (EP + 3) were subjected to sequencing analysis to monitor the dynamics of genomic adaptation to the culture environment. For certain strains, two independent cell cultures were derived for EP + 3 to test for reproducibility of genome adaptation between biological replicates (EP + 3.1 and EP + 3.2). Download FIG S1, PDF file, 0.1 MB. Copyright © 2018 Bussotti et al. 2018 Bussotti et al. This content is distributed under the terms of the Creative Commons Attribution 4.0 International license.

We first used the EP sequence information to confirm species determination and to characterize strain-specific genetic variations that may inform on mechanisms of adaptation. Principal-component analysis (PCA) and clustering analyses based on the average nucleotide identity (ANI) among strains confirmed the molecular determination of the various Leishmania species (see Fig. S2A and B in the supplemental material), with L. infantum and L. donovani or L. major and L. tropica grouping together, respectively. Ldo_CH33 grouped with other L. donovani strains, thus, confirming previous zymodeme analysis (17–19). Based on branch length that correlates with genetic distance, the L. infantum isolates Linf_ZK27, Linf_LLM56, Linf_LLM45, and Linf_02A are highly related, as was expected by their common epidemiological classification as MON-1 (see Table S1 at GitLab).

10.1128/mBio.01399-18.2 FIG S2 Species validation. The genomic distance between the Leishmania isolates used in this study and the indicated Leishmania reference assemblies is shown by the PCA (A) and clustering analyses (B). In the PCA plot, the L. donovani and the L. major clusters are, respectively, highlighted in green and cyan. Download FIG S2, PDF file, 0.5 MB. Copyright © 2018 Bussotti et al. 2018 Bussotti et al. This content is distributed under the terms of the Creative Commons Attribution 4.0 International license.

**Fig. 1:**
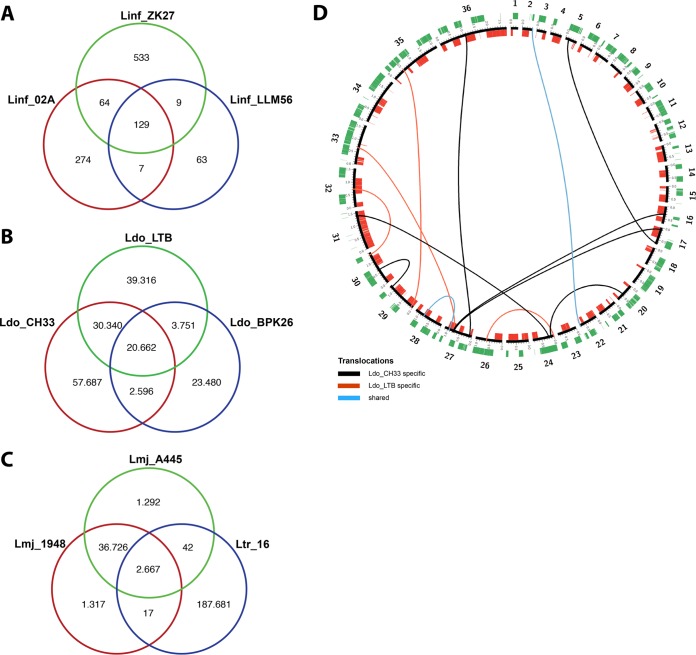
SNVs and translocations with respect to the reference genomes. Venn diagrams show the number of unique and shared SNVs among three L. infantum strains (A), three L. donovani strains (B), and two L. major strains together with an L. tropica strain (C). (D) Circos representation of genomic translocations in samples Ldo_CH33 and Ldo_LTB compared to the corresponding L. donovani reference genome. Connecting lines represent translocations events. Black and red lines demonstrate, respectively, Ldo_CH33 and Ldo_LTB specific translocations. Blue lines show translocations common in both strains. No inversions were detected using the filtering settings indicated in the Materials and Methods section. Black, chromosomes; red, genes mapping on the positive strand; green, genes mapping on the negative-strand.

Comparison of the repertoires of high-frequency single-nucleotide variants (SNVs [>90%]) across the L. infantum isolates (Fig. 1A) confirmed the very close relationship among these samples despite their geographic distance, with less than 600 strain-specific SNVs observed for a given isolate. The majority of SNVs show a low frequency (data not shown), suggesting that nucleotide variants may not be under strong selection in this species. In contrast, the L. donovani strains are evolutionarily more distant, as judged by the presence of over 40,000 strain-specific SNVs, with high-frequency SNVs likely being associated with defined haplotypes that may be under selection, as previously suggested (13, 20), or may be the result of geographic separation and genetic drift (Fig. 1B).

Finally, the SNV analysis revealed the close genetic relationship between the Tunisian and Algerian L. major samples, with 36,726 SNVs shared between the strains compared to the reference genome (Fig. 1C). The massive amount of SNVs identified in L. tropica confirmed the large evolutionary distance to L. major strains observed by PCA and the clustering analyses (Fig. S2). Differences in the evolutionary relationship were further supported by the absence of inversions or translocations in the L. major and L. infantum strains compared to the corresponding reference genomes and the presence of translocations in the Cypriot Ldo_CH33 strain and the Sudanese L. donovani strain Ldo_LTB (Fig. 1D; see Table S6 at GitLab [https://gitlab.pasteur.fr/gbussott/Leishmania_genome_dynamics_during_environmental_adaptation_reveals_strain_specific_differences/]), revealing a potential role of these structural genome variations in L. donovani adaptation.

### Strain-specific gene copy number variations.

**Fig. 2:**
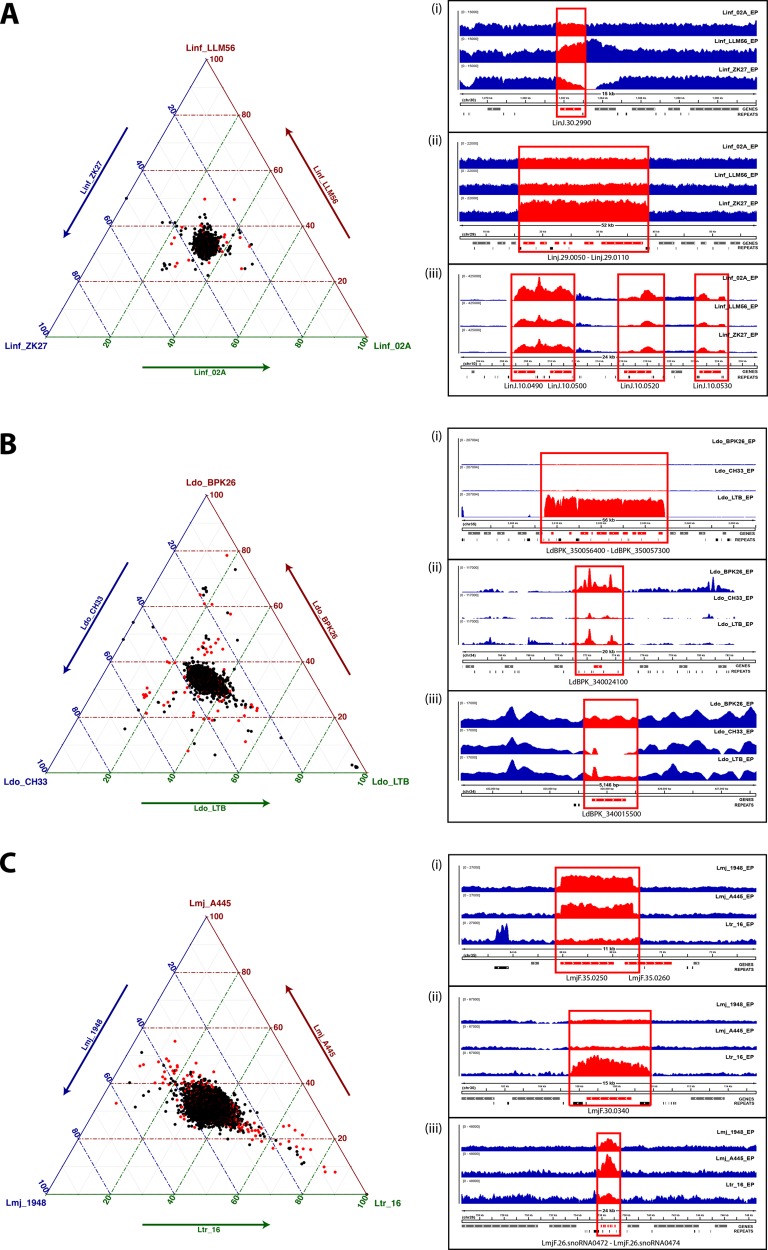
Interstrain gene CNV. (A to C) Ternary plots showing for each gene the relative abundance in the three considered strains (left panels). The axes report the fraction of the normalized gene coverage in the three strains, with each given point adding up to 100. Black dots represent unique genes, whereas red dots indicate genes representing gene families. Comparisons of three L. infantum strains (A), three L. donovani strains (B), and two L. major strains together with an L. tropica strain (C) are shown. The right panels show examples of detected gene copy number variations (CNVs). From top to the bottom, the tracks represent the sequencing depth measured in the three strains, the gene annotations, and the predicted repetitive elements. Coverage tracks were produced with bamCoverage from the deepTools suite (48) (version 2.4.2), ignoring duplicated reads. Normalization of reads per kilobase per million (RPKM) was applied to render the coverage comparable across samples.

Cross-comparison of read depths among the EP samples revealed important intraspecies variations in copy number for single- and multicopy genes (see Materials and Methods and see Table S7 at GitLab [https://gitlab.pasteur.fr/gbussott/Leishmania_genome_dynamics_during_environmental_adaptation_reveals_strain_specific_differences/]). Plotting the gene coverage values for the three L. infantum isolates, the three L. donovani isolates, or the two L. major isolates together with the L. tropica sample, resulted in strong, confined signals at the center of the ternary plots that correspond to genes with equal copy number and thus a 33% distribution across the three axes (Fig. 2, left panels). Compared to the different reference genomes, we observed important, strain-specific differences in gene copy number that are visualized on these plots by shifts of the signals out of the center. Overall, using a cutoff of a 0.5 increase or decrease in a normalized read depth of 1 (corresponding to the copy number per haploid genome), we observed 67, 152, and 119 strain-specific amplifications, respectively, for L. infantum, L. donovani, and L. major (see Table S8 at GitLab). A selection of annotated genes is shown in Table 1 and Table 2 (for the full panel, see Table S8), and prominent examples are represented in the right panels of Fig. 2.

**TABLE 1:** Selection of gene CNVs in *L. infantum* field isolates^*a*^

Gene_id^*b*^	Normalized mean read depth of:			Annotation
	Linf_ZK27	Linf_LLM56	Linf_02A	
LinJ.08.0780	0.96	1.12	2.18	Amastin-like protein
LinJ.09.0200	5.72	9.86	8.1	Putative ATG8/AUT7/APG8/PAZ2
LinJ.10.0490*	18.1	20.55	32.92	GP63, leishmanolysin
LinJ.12.0661	11.63	13.46	6.1	Conserved hypothetical protein
LinJ.15.1240	1.96	3.82	3.87	Putative nucleoside transporter 1
LinJ.19.0820	9.58	14.39	9.09	Putative ATG8/AUT7/APG8/PAZ2
LinJ.23.1330	2.45	3.44	1.46	Hypothetical protein, unknown function
LinJ.26.snoRNA1	3.25	3.77	4.91	ncRNA^*c*^
LinJ.26.snoRNA15	4.2	4.74	6.21	ncRNA
LinJ.26.snoRNA2	3.59	4.34	5.51	ncRNA
LinJ.26.snoRNA3	3.92	4.67	6.04	ncRNA
LinJ.26.snoRNA4	4.03	5	6.28	ncRNA
LinJ.26.snoRNA5	3.94	4.94	6.2	ncRNA
LinJ.26.snoRNA6	4.41	5.04	6.61	ncRNA
LinJ.26.snoRNA7	4.64	5.18	6.9	ncRNA
LinJ.29.0060*	2.04	1.08	0.96	Putative tryptophanyl-tRNA synthetase
LinJ.29.0070*	2.17	1.02	1.01	QA-SNARE protein putative
LinJ.29.0080*	2.07	1.08	0.99	Conserved hypothetical protein
LinJ.29.0090*	2.09	1.03	1.05	Putative Ras-like small GTPases
LinJ.29.1610	1.89	4.45	1.81	Conserved hypothetical protein
LinJ.29.2570	3.2	2.41	1.92	Putative 60S ribosomal protein L13
LinJ.30.2990*	0.98	3.57	2.01	G3P dehydrogenase
LinJ.31.1470	1.98	1.96	1.17	Hypothetical protein, unknown function
LinJ.31.1930	10.41	16.79	15.38	Ubiquitin-fusion protein
LinJ.31.2390	1.04	1.04	0	Helicase-like protein
LinJ.33.0360	20.87	13.19	12.22	Heat shock protein 83-1
LinJ.34.1020	2.11	1.22	2.16	Putative amastin-like surface protein
LinJ.34.1680	4.07	6.09	3.99	Putative amastin-like surface protein
LinJ.36.0190	3.1	5.62	7.22	Elongation factor 2

a For full data, see Table S7 at GitLab (https://gitlab.pasteur.fr/gbussott/Leishmania_genome_dynamics_during_environmental_adaptation_reveals_strain_specific_differences/).

b Asterisks indicate genes shown in the right panel of Fig. 2.

c ncRNA, noncoding RNA.

**TABLE 2:** Selection of gene CNVs in *L. donovani* field isolates^*a*^

Gene_id^*b*^	Normalized mean read depth of:			Annotation
	Ldo_CH33	Ldo_BPK26	Ldo_LTB	
LdBPK_040006600	6.17	0.94	4.8	Hypothetical protein, conserved
LdBPK_050017700	14.07	12.32	9.35	snoRNA
LdBPK_080012500	10.68	9.38	7	Amastin-like protein
LdBPK_080013600	7.46	4.69	4.1	Amastin-like protein
LdBPK_080015900	7.21	10.48	6.93	Cathepsin L-like protease
LdBPK_090006900	8.63	4.22	9.44	Putative ATG8/AUT7/APG8/PAZ2
LdBPK_100009300	4.49	15.24	5.36	Folate/biopterin transporter, putative
LdBPK_120013500	10.18	7.52	18.83	Surface antigen protein 2, putative
LdBPK_120014600	18.73	8.8	15.23	Hypothetical protein
LdBPK_190014300	11.45	7.24	13.77	Putative ATG8/AUT7/APG8/PAZ2
LdBPK_270021500	2.11	4.16	3.06	Amino acid transporter, putative
LdBPK_270026500	3.24	1.13	5.69	Amino acid aminotransferase, putative
LdBPK_270030100	21.94	10.67	6.68	18S, ribosomal, SSU, RNA
LdBPK_270030130	20.81	10.7	6.4	rRNA
LdBPK_270030140	21.2	10.73	6.74	28S, ribosomal, RNA, LSU-α
LdBPK_270030150	19.96	9.97	6.18	28S, ribosomal, RNA, LSU-β
LdBPK_270030160	17.77	9.65	5.93	28S, ribosomal, RNA, LSU-δ, M2
LdBPK_270030170	21.2	10.74	6.19	28S, ribosomal, RNA, LSU-ζ, M6
LdBPK_270030180	17.68	10.16	5.37	28S, ribosomal, RNA, LSU-ε, M4
LdBPK_280010700	3.08	1.01	2.48	Major surface protease gp63, putative
LdBPK_280035000	8.59	14.66	8.04	Heat shock protein hsp70, putative
LdBPK_300020900	2.34	7.56	1.88	p1/s1 nuclease
LdBPK_310009700	7.22	10.63	6.01	Amastin, putative
LdBPK_310016700	4.3	8.48	5.34	Sodium stibogluconate resistance protein
LdBPK_320043700	3.28	2.02	5.44	HIBCH-like protein
LdBPK_330008700	8.56	13.64	7.76	Heat shock protein 83-17
LdBPK_340015500*	0.07	1.18	0.36	Amastin-like surface protein, putative
LdBPK_340015600	3.19	5.12	3.15	Amastin-like surface protein, putative
LdBPK_340015800	1.78	0.92	3.36	Amastin-like surface protein, putative
LdBPK_340017400	2.75	1.04	0.8	Amastin-like surface protein, putative
LdBPK_340023500	3.03	1.87	9.92	Amastin-like surface protein, putative
LdBPK_340024100*	1.47	26.05	5.71	Amastin surface glycoprotein, putative
LdBPK_350056400*	1	1	48.78	Hypothetical protein
LdBPK_350056500*	1.02	1.07	47.88	Hypothetical protein, conserved
LdBPK_350056600*	1.04	0.98	44.76	Protein-only RNase P, putative
LdBPK_350056700*	1.22	1.1	36.57	Ribosomal protein L37e, putative
LdBPK_350056800*	1.03	1.03	43.11	RNA pseudouridylate synthase, putative
LdBPK_350056900*	1.01	0.91	45.34	Hypothetical protein
LdBPK_350057000*	0.92	0.96	41.41	Hypothetical protein
LdBPK_350057100*	1.05	0.87	42.65	Hypothetical protein, unknown function
LdBPK_350057200*	0.97	0.96	43.22	Biopterin transporter, putative
LdBPK_350057300*	1.06	0.89	44	Hypothetical protein

a For full data, see Table S7 at GitLab (https://gitlab.pasteur.fr/gbussott/Leishmania_genome_dynamics_during_environmental_adaptation_reveals_strain_specific_differences/). PacBio *L. donovani* LDBPK assembly and annotations were downloaded on 02/05/2017 (ftp://ftp.sanger.ac.uk/pub/project/pathogens/Leishmania/donovani/LdBPKPAC2016beta).

b Asterisks indicate genes shown in the right panel of Fig. 2.

In L. infantum, we observed (i) a 2.94-fold amplification in Linf_LLM56 of LinJ.30.2990 encoding a glyceraldehyde 3-phosphate dehydrogenase, (ii) a cluster of seven genes (Linj.29.0050 to Linj.29.0110) located in an ∼23-kb region delimited by SIDER repetitive elements that showed a 2-fold amplification in Linf_ZK27, and (iii) the amplification (up to 32-fold) of the GP63 leishmanolysin cluster (LinJ.10.0490 to LinJ.10.0530) in Linf_02A. For L. donovani, we identified (i) a 48-fold amplification specific to Ldo_LTB of a cluster of 10 genes (LdBPK_350056400 to LdBPK_350057300), which includes a biopterin transporter, an RNase P, an RNA pseudouridylate synthase, and a putative ribosomal L37e protein, (ii) an up to 26-fold amplification in Ldo_BPK26 of a putative amastin surface glycoprotein (LdBPK_340024100), and (iii) the deletion in Ldo_CH33 and partial depletion in Ldo_LTB of a putative amastin-like surface protein (LdBPK_340015500). Finally, as expected from their phylogenetic relationship, important differences were observed in gene CNVs between the L. tropica and L. major strains, including (i) an amplification on chromosome 35 in both Lmj_1948 and Lmj_A445 (respectively, 3.51- and 2.63-fold), spanning a hypothetical protein (LmjF.35.0250) and the 5′ portion of a putative GTPase-activating protein (LmjF.35.0260), (ii) an up to 6-fold amplification in Ltr_16 of a putative KU80 protein (LmjF.30.0340) flanked by SIDER2 elements, and (iii) an Lmj_A445-specific amplification of a small nucleolar RNA (snoRNA) cluster on chromosome 26.

Together these results suggest that gene CNVs may drive or be the result of adaptation of otherwise highly related Leishmania field isolates, causing phenotypic differences with respect to stress resistance, nutrition, and infectivity, as judged by gene CNVs observed in heat shock proteins, transporters, and known virulence factors (Table 1 and Table 2). Thus, gene CNV seems to shape the parasite genome and likely its pathogenic potential in the field through positive (amplification) and purifying (deletion) selection, potentially driving long-term adaptation to ecological constraints of local transmission cycles.

### Dynamic karyotype changes during extended growth in culture.

**Fig. 3:**
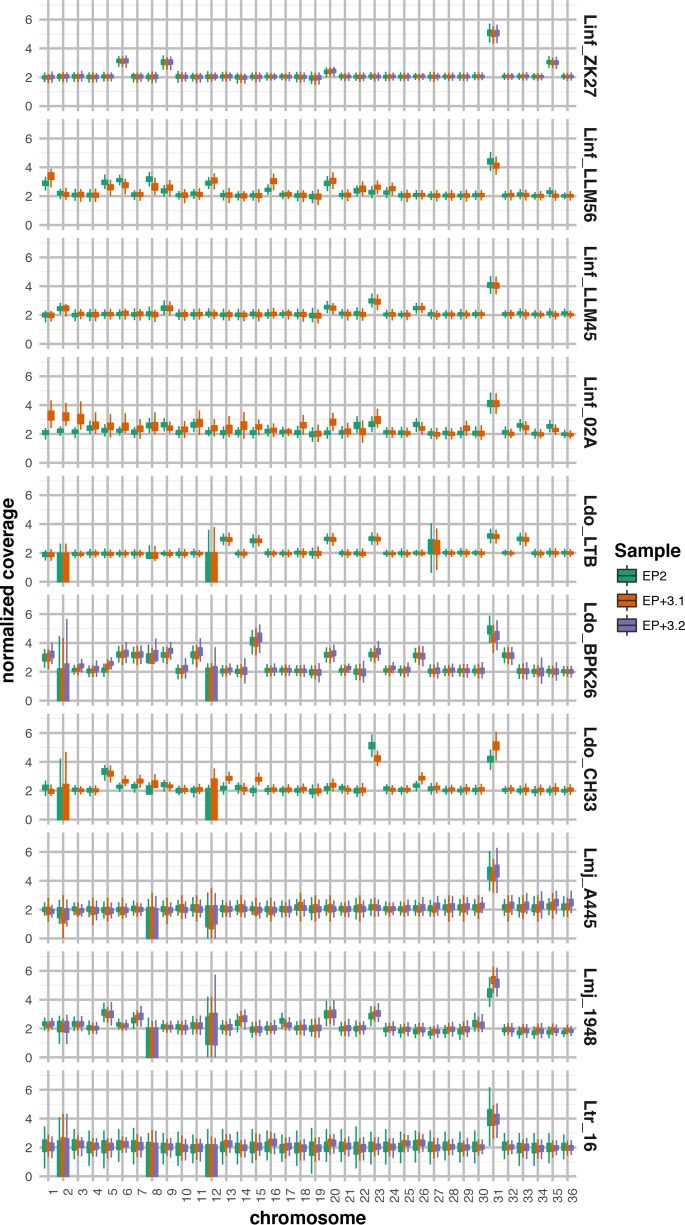
Chromosome ploidy analysis. Box plots represent the normalized sequencing coverage distributions for each chromosome for the strains indicated. The lower and upper edges of the box show, respectively, the lower quartile (i.e., 25% of nucleotides with normalized coverage below that value) and upper quartile (i.e., 25% of nucleotides with normalized coverage above that value). The whiskers show maximum and minimum coverage values, excluding outliers. Outliers are not shown to ease plot readability. Box sizes reflect coverage dispersion that can be affected by sample sequencing depth, chromosomal ploidy, intrachromosomal copy number alterations, assembly gaps, or repetitive regions. The increased box size visible in chromosome 27 of sample Ldo_LTB is caused by a large subchromosomal amplification (Fig. S3). In L. donovani, L. major, or L. tropica samples, the presence of large gaps or repetitive regions inflates the box size for chromosomes 2, 8, and 12. Green, early passage (EP); orange, EP + 3.1 replicate; purple, EP + 3.2 replicate.

We next assessed structural genomic variations that may drive short-term environmental adaptation comparing EP and EP + 3 samples that evolved *in vitro* during culture adaptation. Whole-genome sequencing (WGS) and read depth analysis revealed important karyotype differences between the two *in vitro* passages of a given strain (intrastrain variation) and among different strains (interstrain variation). Aside from an intrachromosomal duplication at both EP and EP + 3 observed in Ldo_LTB spanning nearly half of chromosome 27 (453,410 bases) affecting 113 genes, changes in read depth were homogenous across all chromosomes, thus revealing frequent aneuploidy (see Fig. S3 in the supplemental material). Linf_ZK27 and Ldo_LTB displayed the most stable karyotypes between EP and EP + 3. As judged by read depth values corresponding to integer or intermediate chromosome copy number values, full or mosaic aneuploidy was observed for four chromosomes in Linf_ZK27 (chromosomes 6, 9, 31, and 35) and six chromosomes in Ldo_LTB (chromosomes 13, 15, 20, 23, 31, and 33), which were established at EP and maintained at EP + 3 (Fig. 3; see Table S4 at GitLab [https://gitlab.pasteur.fr/gbussott/Leishmania_genome_dynamics_during_environmental_adaptation_reveals_strain_specific_differences/]). All other isolates showed higher intrastrain karyotype instability with both gain and loss of chromosomes observed between EP and EP + 3. Linf_02A represented the most extreme example showing significant changes in read depth for 21 chromosomes (Fig. 3; see Table S4 at GitLab) and five chromosomes with a somy score difference higher than 0.5 compared to the disomic state corresponding to 2 (see Material and Methods and see Table S4 at GitLab). Overall, chromosomes 20 and 23 showed the highest propensity for amplification between EP and EP + 3, with different ploidy levels (mosaic aneuploidy, trisomy, and tetrasomy) observed in, respectively, 19 and 15 samples out of 25, suggesting that amplification of these chromosomes may provide fitness advantage during culture adaptation for most of the strains analyzed in our study.

10.1128/mBio.01399-18.3 FIG S3 Chromosome coverage analysis. (A) Circos plot representing the normalized sequencing coverage of the strains indicated. The bar height correlates with sequencing coverage. The coverage is shown on the vertical axis and ranges from 0 to 3. The ticks, scaled to represent 100 kb, show the genomic position. Green, early passage (EP); orange, EP + 3.1 replicate; purple, EP + 3.2 replicate. (B) Enlargement of Lmj_1948 chromosomes 10, 11, 14, 24, 26, 27, and 35. Download FIG S3, JPG file, 2.8 MB. Copyright © 2018 Bussotti et al. 2018 Bussotti et al. This content is distributed under the terms of the Creative Commons Attribution 4.0 International license.

With the exception of the previously reported stable aneuploidy for chromosome 31 (10), the dynamics of the observed karyotypic changes are substantially different among all isolates. It is interesting to speculate that this heterogeneity reflects individualized solutions driving fitness gains *in vitro*. While differences in culture conditions certainly account for some of the observed karyotypic variability, the comparison of two closely related Spanish L. infantum isolates, Linf_LLM45 and Linf_LLM56, reveals a culture-independent component implicated in genomic adaptation. Both isolates were adapted to culture at the same time under the same conditions, yet they showed important differences in karyotype dynamics, with only Linf_LLM56 demonstrating changes in somy levels at EP + 3 (Fig. 3; see Table S4 at GitLab [https://gitlab.pasteur.fr/gbussott/Leishmania_genome_dynamics_during_environmental_adaptation_reveals_strain_specific_differences/]). These strains are genotypically identical (zymodeme MON-1) (see Table S1 at GitLab) and are genetically closely related, with an average nucleotide identity of over 99.95%, suggesting that minor genetic differences may have an important impact on Leishmania karyotypic adaptation to a given environment. Aside from SNVs (Fig. 1), the difference in karyotype dynamics may be linked to gene CNVs observed between Linf_LLM45 and Linf_LLM56, which affected genes implicated, for example, in protein translation, protein folding, or protein turnover (Table 3).

**TABLE 3:** Gene CNVs in the Spanish *L. infantum* isolates Linf_LLM45 and Linf_LLM56

Gene	Normalized mean read depth of:		Ratio	Delta	Annotation
	Linf_LLM45	Linf_LLM56			
LinJ.02.0690	1.6	2.1	0.7	0.5	Hypothetical protein, unknown function
LinJ.03.0420	1.4	1.9	0.7	0.6	Putative 60S acidic ribosomal protein P2
LinJ.04.0160	1.4	2.0	0.7	0.6	Hypothetical protein
LinJ.04.0180	2.2	1.1	2.0	1.1	Surface antigen-like protein
LinJ.05.snoRNA3	7.9	8.4	0.9	0.6	ncRNA^*a*^
LinJ.05.snoRNA5	7.7	8.8	0.9	1.1	ncRNA
LinJ.09.0200	8.8	7.8	1.1	1.0	ATG8/AUT7/APG8/PAZ2, cytoskeleton
LinJ.10.0490	15.4	16.7	0.9	1.3	GP63, leishmanolysin
LinJ.11.1110	3.3	1.9	1.7	1.4	Putative 60S ribosomal protein L28
LinJ.11.1120	2.1	1.0	2.1	1.1	Conserved hypothetical protein
LinJ.13.0330	11.3	10.0	1.1	1.3	α-Tubulin
LinJ.14.0400	1.8	3.8	0.5	2.0	Conserved hypothetical protein
LinJ.15.snoRNA4	15.3	13.8	1.1	1.5	ncRNA
LinJ.17.0090	21.1	21.8	1.0	0.8	Elongation factor 1-α
LinJ.18.1500	4.0	3.1	1.3	0.9	Putative P-type H^+^-ATPase
LinJ.19.0820	9.9	11.3	0.9	1.4	Putative ATG8/AUT7/APG8/PAZ2
LinJ.19.1350	2.7	3.8	0.7	1.0	Putative glycerol uptake protein
LinJ.22.snoRNA1	5.7	4.7	1.2	1.0	ncRNA
LinJ.26.snoRNA10	5.4	4.9	1.1	0.5	ncRNA
LinJ.26.snoRNA15	5.4	4.7	1.1	0.6	ncRNA
LinJ.26.snoRNA7	5.8	5.2	1.1	0.7	ncRNA
LinJ.29.1570	1.0	1.6	0.7	0.5	Conserved hypothetical protein
LinJ.29.1580	1.0	1.5	0.7	0.5	Conserved hypothetical protein
LinJ.29.1610	2.8	3.7	0.8	0.9	Conserved hypothetical protein
LinJ.29.2240	1.2	1.8	0.6	0.6	Conserved hypothetical protein
LinJ.30.0690	3.6	3.0	1.2	0.6	Putative 40S ribosomal protein S30
LinJ.30.1660	2.0	1.4	1.4	0.6	Conserved hypothetical protein
LinJ.30.3550	1.0	2.0	0.5	1.0	Conserved hypothetical protein
LinJ.30.3560	1.0	2.0	0.5	1.0	*S*-Adenosylmethionine synthetase
LinJ.31.0460	3.0	1.0	2.9	2.0	Putative amastin
LinJ.31.1660	2.9	2.1	1.4	0.8	3-Ketoacyl-CoA thiolase-like protein
LinJ.31.1930	16.1	13.4	1.2	2.7	Ubiquitin-fusion protein
LinJ.32.1910	2.8	1.8	1.6	1.0	Putative iron superoxide dismutase
LinJ.33.0360	5.8	11.3	0.5	5.6	Heat shock protein 83-1
LinJ.34.1010	5.4	3.8	1.4	1.6	Putative amastin-like surface protein
LinJ.34.1020	3.1	1.2	2.6	1.9	Putative amastin-like surface protein
LinJ.34.1680	4.1	6.1	0.7	2.0	Putative amastin-like surface protein
LinJ.34.1730	10.9	14.4	0.8	3.5	Putative amastin-like surface protein
LinJ.36.0190	6.0	5.0	1.2	1.0	Elongation factor 2
LinJ.36.1680	1.8	2.5	0.7	0.6	Universal minicircle sequence bd. protein
LinJ.36.3010	1.5	2.3	0.7	0.8	40S ribosomal protein S24e

a ncRNA, noncoding RNA.

Despite this remarkable plasticity of the Leishmania karyotype, we observed that changes in chromosome number are highly reproducible in duplicate EP + 3 samples that were derived for L. major (Lmj_1948 and Lmj_A445), L. infantum (Linf_ZK27), L. donovani (Ldo_BPK26), and L. tropica (Ltr_16) (Fig. 3). Thus, even though karyotypic fluctuations may arise in a stochastic manner—either in the host or during culture adaptation—our data demonstrate that beneficial karyotypes are under strong selection during culture adaptation. Significantly, the SNV frequency profiles for EP and EP + 3 were largely identical, ruling out the possibility that adaptation occurs through selection of subpopulations that would cause important shifts in SNV frequency distribution (data not shown). Together our results document the highly dynamic nature of karyotype management in Leishmania during environmental adaptation that is likely governed by complex interactions between external cues and intrinsic genetic differences.

### Dynamic variations in gene copy number during *de novo* culture adaptation.

**Fig. 4:**
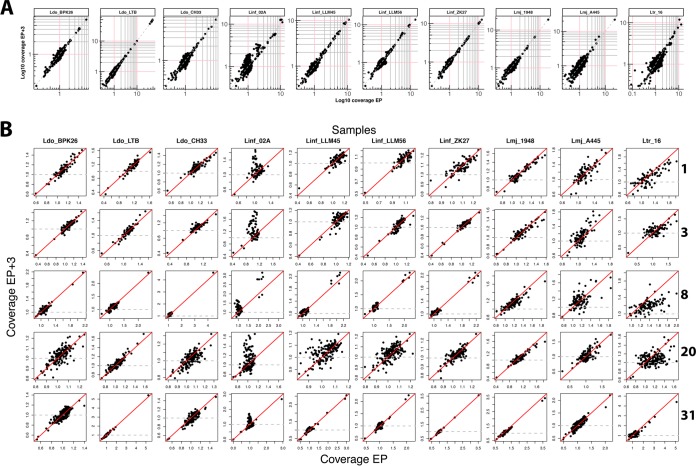
Gene copy number variation (CNV) during culture adaptation. (A) Genome-wide scatter plot showing log_10_ gene coverage of EP and EP + 3 samples. Dots represent all genes annotated in the respective reference assemblies. (B) Chromosome-specific scatter plots of gene CNVs between EP + 3 versus EP. Only selected chromosomes are shown, and the full panel is available in Fig. S4. The red diagonal lines indicate the bisectors. The gray dashed horizontal lines mark a coverage value of 1. The axes’ maximum and minimum values were adjusted to the most extreme values for each plot to avoid logarithmic compression. For both panels A and B, the EP + 3.1 replicate was used, except for Lmj_A445, for which the EP + 3.2 replicate was utilized.

Plotting of genome-wide sequencing coverage of EP + 3 against EP for all annotated genes resulted in a largely diagonal distribution, suggesting that there are no major CNVs between the two different passages (Fig. 4A; see Fig. S4 in the supplemental material and see Table S9 at GitLab [https://gitlab.pasteur.fr/gbussott/Leishmania_genome_dynamics_during_environmental_adaptation_reveals_strain_specific_differences/]). Overall, the majority of genes were scattered around a normalized coverage of 1 (corresponding to the copy number per haploid genome [see Materials and Methods]), suggesting that their copy number matches the one in the reference strains. We nevertheless observed a significant number of genes across all isolates that showed coverage either below 0.5-fold or above 2-fold, independent of culture passage, thus, revealing important differences between the isolates and their corresponding reference genomes. This analysis uncovered a significant increase in coverage at EP + 3 for all chromosomes of strain Linf_02A (Fig. 4B; see Table S9 at GitLab), indicating some form of CNV that correlated with increased culture passage. In the following analyses, we more closely investigated the structural basis of these culture-associated CNVs in Linf_02A.

10.1128/mBio.01399-18.4 FIG S4 Chromosome-specific gene coverage variation analysis. For each sample and for each chromosome, the scatter plots show the normalized gene coverage for EP + 3 (*y* axis) versus EP (*x* axis). The red diagonal lines indicate the bisectors. To show the extent of gene CNV with respect to the reference genomes, the axis limits are not fixed but dynamically assigned for each chromosome to include the maximum and the minimum measured values. Download FIG S4, PDF file, 1.6 MB. Copyright © 2018 Bussotti et al. 2018 Bussotti et al. This content is distributed under the terms of the Creative Commons Attribution 4.0 International license.

### Telomeric amplification.

**Fig. 5:**
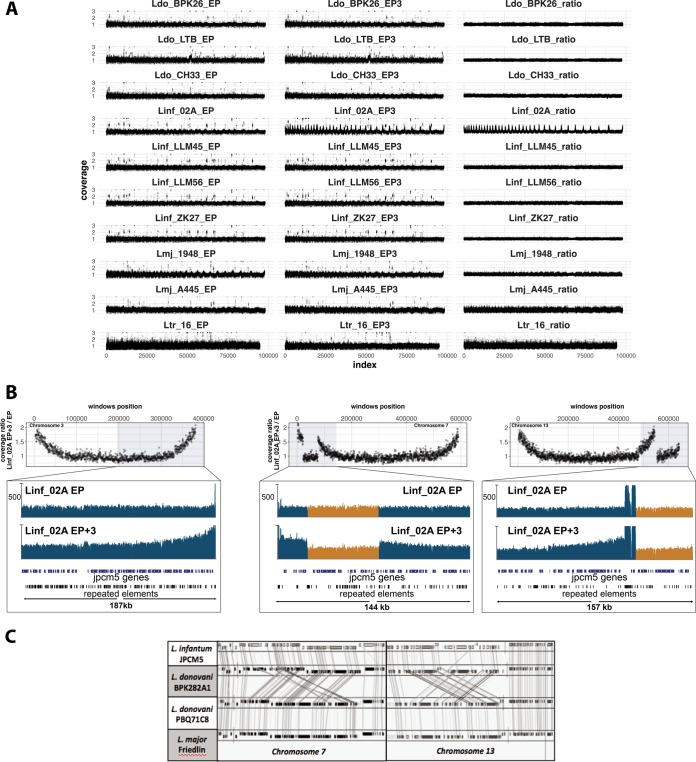
Subtelomeric amplification. (A) Genome-wide coverage ratios (*y* axes) between EP and EP + 3 of the indicated samples and their respective reference genomes (left and middle panels) or between EP + 3/EP (right panels) are shown. The EP + 3 coverage refers to the EP + 3.1 replicate, except for Lmj_A445, for which EP + 3.2 replicate coverage was used. The *x* axis reports the position of the genomic windows along the chromosomes. Dots represent genomic windows of 300 bases. In each panel, the 36 Leishmania chromosomes are shown in sequential order. To ease the visualization, all scores of >3 were assigned to a value of 3. (B) The EP + 3/EP coverage ratio for chromosomes 3, 7, and 13 of sample Linf_02A (top panel) and the Integrative Genomics Viewer (IGV) snapshots of the respective chromosome extremities (bottom panel) are shown. The lower tracks (in order of appearance from the top) correspond to sequencing coverage in EP, sequencing coverage in EP + 3, repeat elements, or predicted low-complexity region predictions and L. infantum gene annotations. The sequencing coverage tracks range from 0 to 500×. For chromosomes 7 and 13, the bottom panels highlight in orange the misassembled regions. (C) SyntView snapshot of chromosomes 7 and 13. From top to bottom, the tracks show the orthologous genes in L. infantum JPCM5, L. donovani BPK282A1, L. donovani PBQ71C8, and L. major Friedlin. Straight lines connect the orthologous genes in different genomes. The diagonal lines are indicative of misassembled genomic regions.

We partitioned the genome into contiguous windows and plotted the coverage at EP or EP + 3, as well as the ratio between EP + 3 and EP. We observed a significant increase in read depth toward the telomeres in both EP and EP + 3 for Lmj_1948, while coverage fluctuations in EP + 3 were observed for Ltr_16, Lmj_A445, and Linf_02A, generating a repetitive pattern when plotting the entire genome (Fig. 5A). The observed increase in read depth is not discrete but gradual, spanning from subtelomeric regions to the telomeres and thus cannot be assigned to misannotation of the number of telomeric repeats in the reference genome. (That should cause a discrete but not progressive increase in read depth at the telomeres only.) The gradual increase in read depth supports the increased gene coverage and contributes to the shift in the chromosome coverage distribution we observed for strain Linf_02A at EP + 3 (Fig. 3 and Fig. 4B). We found the gradual increase in read depth to be disrupted for chromosomes 7 and 13 by regions with lower read depth (Fig. 5B; see Fig. S5 in the supplemental material). According to our model, these genomic elements should not be part of subtelomeric regions and thus either reflect a strain-specific recombination event or misassembly of the L. infantum reference genome. Synteny analysis among available reference genomes showed that the disruptive sequence elements observed in Linf_02A show subtelomeric localization in L. major and the novel PacBio-generated LdBPK genome (12), revealing misassembly of these regions in the current L. infantum and the previous L. donovani reference genomes (Fig. 5C). This “diagnostic” value of our result confirms that telomeric amplification is not a technical artifact but represents a nonconventional mechanism of telomeric amplification in Leishmania that may be similar to those described in other organisms (21).

10.1128/mBio.01399-18.5 FIG S5 Chromosome-specific bin coverage variation analysis. Dots represent adjacent genomic intervals of 300 bases. For each sample, separate panels represent different chromosomes. The *x* axis in each panel represents the genomic coordinates, while the *y* axis indicates the normalized sequencing coverage. Intervals with coverage superior to 2 are highlighted in orange, and scores of >3 are assigned to 3. Intervals with coverage lower than 0.5 are highlighted in blue. Download FIG S5, JPG file, 2.7 MB. Copyright © 2018 Bussotti et al. 2018 Bussotti et al. This content is distributed under the terms of the Creative Commons Attribution 4.0 International license.

## DISCUSSION

Drawing from newly generated genome sequences of Leishmania clinical isolates and conducting longitudinal studies *in vitro*, we demonstrate the existence of strain-specific gene copy number variations that may drive long-term and short-term evolutionary trajectories in Leishmania. We show that highly related Leishmania isolates that evolved in different regions are distinguished by both amplification and loss of genes linked to parasite infectivity, such as GP63 or amastins. The fixation of these genetic alterations may not be random but could potentially be the result of positive or purifying selection processes that are functional and adapt parasite fitness to a given ecology or transmission cycle. Identification of such genomic alterations that are under selection by the host can directly inform about genetic loci that are clinically relevant. The corresponding genes may be prioritized for functional genetic analysis (notably those genes that are not annotated) as they may play important roles in virulence and may qualify as biomarkers with diagnostic or prognostic value.

Monitoring of genetic fluctuations using *de novo* culture as a proxy for short-term environmental adaptation revealed two forms of dynamic genomic changes. First, as judged by the establishment of reproducible aneuploidy profiles in duplicate cultures of a given strain, chromosomal amplification is the result of selection rather than random genetic drift. This result corroborates our previous observations in the L. donovani experimental strain LD1S, where spontaneous karyotypic fluctuations generate genotypically and phenotypically diverse mosaic populations that are substrates for evolutionary adaptation and fitness gain in response to environmental change (13). Whether chromosomal amplification occurs *de novo* during culture adaptation or reflects an initial diversity in each clinical isolate remains to be established, even though the karyotype mosaicism we previously observed *in situ* in L. donovani-infected hamster spleen and liver favors the latter explanation (13).

Second, we uncovered a novel mechanism of telomeric amplification in three different Leishmania species (L. major, L. tropica, and L. infantum), as revealed by a progressive increase in sequencing read depth toward the chromosome ends. Nonclassical mechanisms of telomere maintenance have been documented in a variety of eukaryotes, including (i) rolling circle replication in Kluyveromyces lactis, implicating extrachromosomal circular templates (22), (ii) break-induced replication in Saccharomyces cerevisiae involving recombination between tracts of telomeric repeats (23), or (iii) the telomeric loop formation first observed in human and mouse cells, in which a telomere 3′ end loops back to invade the duplex part of the same telomere and anneals with complementary telomeric repeat sequence (21). Our observation of a gradual increase in read depth from large subtelomeric regions toward the chromosome ends is compatible with rolling circle replication, considering the propensity of Leishmania to extrachromosomal amplification (9), the absence of telomeric repeats in subtelomeric regions in Linf_02A that would allow for telomeric loop formation (data not shown), and the presence of only very small telomeric loops of less than 1 kb in the related pathogen Trypanosoma brucei (24). Given that bona fide amastigotes cannot be maintained or adapted to culture, our *in vitro* evolutionary experiments were conducted with insect-stage promastigotes that were directly derived from tissue-derived amastigotes. Thus, the various forms of genomic instability we observed in our system likely drive adaptation and fitness gain in the sand fly vector. While we previously documented the prevalence of chromosomal amplification in tissue amastigotes (13), the presence of telomeric amplification at this stage remains to be established.

Our comparative genomics approach further provided a powerful tool to reveal species- and strain-specific variations in genomic adaptation. Telomeric amplification was only seen in 3 of the 10 isolates, and very different karyotypic solutions were observed even in closely related isolates under the same culture conditions, revealing the significance of environment-independent, intrinsic factors in genomic adaptation. Using the highly related Spanish isolates Linf_LLM56 and Linf_LLM45 as an example, various genetic determinants may be implicated. Both strains were obtained from the same area in a short time frame, suggesting a very recent common ancestor, as confirmed by their genetic similarity. Nevertheless, they were isolated from two stray dogs, and genetic differences of both mammalian and insect hosts during natural infection may have shaped the parasite genomes in different ways through genotype-genotype interactions, as observed, for example, in anopheline mosquitoes infected with Plasmodium falciparum, the causal agent of malaria (25). Given the intrinsic instability of the Leishmania karyotype we observed *in situ* during visceral infection in liver- and spleen-derived amastigotes (13), these interactions may establish a very different chromosomal stoichiometry among canine isolates, which then translates into the different karyotypic trajectories we observed during culture adaptation. Likewise, differences in the number of single-copy genes or CNVs in multicopy gene arrays generated by intra- or extrachromosomal amplification (9) may impact the karyotypic profile, with gene amplification alleviating the need for chromosome duplication as previously suggested (10). Finally, we cannot rule out that individual SNVs in coding sequences or regulatory element 5′ and 3′ untranslated regions (UTRs) may have an impact on genomic adaptation, a possibility that is supported by our previous observation of tissue-specific haplotype selection in the liver and spleen of L. donovani-infected hamsters (13).

In conclusion, our results draw a complex picture of Leishmania genomic adaptation in the field and in culture that needs to be considered in epidemiological studies that correlate parasite phenotypic variability and disease outcome. Adaptation is highly individualized and results from a dynamic selection process acting on genetically heterogeneous parasite populations that thrive inside distinct and genetically equally heterogeneous hosts (e.g., insects, rodents, and humans). For environmental adaptation, Leishmania can draw from a vast genetic landscape of spontaneous karyotypic fluctuations, stochastic gene amplifications, and nucleotide polymorphisms. Our comparison of highly related Spanish L. infantum isolates revealed that even small variations in sequence might result in important differences in karyotypic adaptation. Thus, closely related isolates evolving in the same epidemiological niche can attain similar levels of fitness in a highly pleiotropic way using alternative genetic solutions (13). This form of pleiotropic adaptation is characteristic for pathogenic microbes that maintain genetic heterogeneity, and thus evolvability, despite strong selection. Our data indicate that Leishmania adopts a similar, polyclonal adaptation strategy, which may strongly limit the identification of biomarkers with broad clinical relevance across Leishmania species or even related Leishmania strains. Future efforts need to take this complexity into account and approach the epidemiology of Leishmania infection on an integrative level, considering genotype-genotype and environment-genotype interactions and dissecting the population structure of individual isolates by single-cell, direct tissue sequencing.

## MATERIALS AND METHODS

### *Leishmania* parasite isolation and culture.

Ten Leishmania strains belonging to the L. tropica, L. major, and L. donovani complexes of eight different geographical areas were isolated from infected patients, dogs, or hamsters (see Table S1 at GitLab [https://gitlab.pasteur.fr/gbussott/Leishmania_genome_dynamics_during_environmental_adaptation_reveals_strain_specific_differences/]). Some strains were cryopreserved in liquid nitrogen prior to culture adaptation until used for this study (see Table S1 at GitLab). Leishmania isolates were first stabilized *in vitro* in media that were optimized in the various LeiSHield partner laboratories (stabilization medium; see Table S2 at GitLab), prior to expansion in classical RPMI culture medium for a defined number of passages (expansion medium). Seven strains belonging to the L. donovani complex were selected for the comparison of intraspecies evolvability in culture. These include the four L. infantum strains Linf_ZK27 from Tunisia, Linf_LLM56 and Linf_LLM45 from Spain, and Lin_02A from Brazil (voucher to assess this sample at Coleção de Leishmania do Instituto Oswaldo Cruz [CLIOC]: IOCL3598), as well as the three L. donovani strains Ldo_BPK26 from India, Ldo_LTB from Sudan, and Ldo_CH33 from Cyprus. The latter strain belongs to the L. donovani MON-37 zymodeme (17–19), and multilocus microsatellite typing (MLMT) analysis has positioned it in a novel L. donovani *sensu lato* (s.l.) group (26). Our analysis further included two L. major strains (Lmj_1948 from Tunisia, Lmj_A445 from Algeria) and one L. tropica strain (Ltr_16 from Morocco) (see Table S1 at GitLab). Genotyping methodologies were applied to confirm species identity of the strains used in this work (see Table S1 at GitLab). Standardized procedures for DNA sample preparation and cell culturing or subculturing were used in all partner laboratories (see Table S2 at GitLab). Promastigotes from early cell culture (passage 2 of growth in expansion medium, referred to as early passage [EP] samples) and derived parasites maintained in culture for three more *in vitro* passages (EP + 3) were processed for whole-genome sequencing (WGS) using parasites from the late logarithmic growth phase. While different Leishmania strains can show differences in terms of generation time and can reach different population densities, we previously estimated that a single passage in culture corresponds to ca. 10 generations (13). To determine reproducibility of *in vitro* genome evolution, duplicate EP + 3 samples (EP + 3.1 and EP + 3.2) were generated for the Linf_ZK27, Lmj_1948, Lmj_A445, Ldo_BPK26, and Ltr_16 strains (Fig. S1). Culture conditions and time in culture for the 25 samples are detailed in Table S2 at GitLab.

### Nucleic acid extraction, sample preparation, and sequencing analysis.

Procedures for DNA sample preparation and quality control were standardized using common protocols. Briefly, DNA extraction was performed using DNeasy blood and tissue kits from Qiagen according to the manufacturer’s instructions. Nucleic acid concentrations were measured with Qubit, and the DNA quality was evaluated on agarose gel. Between 2 and 5 µg of DNA was used for sequencing. The following samples showed small DNA amounts and were thus PCR amplified before sequencing: Ldo_LTB_EP (5 cycles), Ldo_LTB_EP + 3 (5 cycles), Linf_02A_EP (10 cycles), Linf_02A_EP + 3 (5 cycles). No PCR amplification was performed for the other samples.

Whole-genome, short-insert, paired-end libraries were prepared for each sample. Samples Ltr_16_EP, Ltr_16_EP + 3.1, Ltr_16_EP + 3.2, Ldo_BPK26_EP, Ldo_BPK26_EP + 3.1, Ldo_BPK26_EP + 3.2, Lmj_A445_EP, Lmj_A445_EP + 3.1, and Lmj_A445_EP + 3.2 were sequenced by the Biomics sequencing platform (https://research.pasteur.fr/en/team/biomics/) with Hiseq 2,500 rapid runs, resulting in 2 × 108-bp reads using the NEXTflex PCR-Free kit. All other samples were sequenced with the KAPA Hyper Prep kit (Kapa Biosystems) at Centro Nacional de Análisis Genómico (CNAG [http://www.cnag.crg.eu/]) using the TruSeq SBS kit v3-HS (Illumina, Inc.). Multiplex sequencing was performed according to standard Illumina procedures, using HiSeq2000 flowcell v3, generating 2 × 101-bp paired-end reads.

### Read alignment.

Gene annotations and reference genomes of L. major Friedlin and L. infantum JPCM5 were downloaded from the Sanger FTP server (27). (ftp://ftp.sanger.ac.uk/pub/project/pathogens/gff3/CURRENT/) on 5 September 2017, whereas PacBio L. donovani LDBPK assembly and annotations were downloaded on 5 February 2017 (ftp://ftp.sanger.ac.uk/pub/project/pathogens/Leishmania/donovani/LdBPKPAC2016beta). The reads were aligned to the reference genomes with BWA mem (version 0.7.12) (28, 29) with the flag -M to mark shorter split hits as secondary. Samtools fixmate, sort, and index (version 1.3) (30) were used to process the alignment files and turn them into bam format. RealignerTargetCreator and IndelRealigner from the GATK suite (31–33) were run to homogenize indels. Eventually, PCR and optical duplicates were labeled with Picard MarkDuplicates [version 1.94(1484)] (https://broadinstitute.github.io/picard/) using the option “VALIDATION_STRINGENCY=LENIENT.” While the reads were aligned against full assemblies, including unsorted contigs, just the canonical 36 chromosomes were considered for downstream analyses of ploidy estimation and copy number alterations. This filter was necessary because of the high content of repetitive elements and the absence of comparable and high-quality annotations in the contigs. Given that the L. tropica reference genome is still unfinished, the sample Ltr_16 was aligned against the L. major Friedlin genome. Overall, starting from a total of 1,011,803,806 short reads, 952,093,114 were successfully aligned to the respective reference genomes (see Table S3 at GitLab [https://gitlab.pasteur.fr/gbussott/Leishmania_genome_dynamics_during_environmental_adaptation_reveals_strain_specific_differences/]). Picard CollectAlignmentSummaryMetrics was used to estimate sequencing and mapping statistics.

### Comparative genome analysis.

Whole-genome sequencing data from the EP Leishmania isolates were processed with Trimmomatic (version 0.35) (34) to remove low-quality bases (options “LEADING:3 TRAILING:3 SLIDINGWINDOW:4:15”) and adapter contaminations (option “ILLUMINACLIP,” with values 2:30:12:1:true). Reads that were shorter than 36 bases after filtering were discarded (option “MINLEN:36”). The trimmed reads were assembled with SPAdes (35) (version 3.7.0) with the option “careful.” The resulting contigs were used to estimate the average nucleotide identity (ANI) with the dnadiff part of the MUMmer system (version 3.23) (36). The analysis included the reference genomes of L. donovani, L. infantum, and L. major that were retrieved from the Sanger database (described above) and reference genomes of L. braziliensis, L. mexicana, and L. panamensis that were retrieved from ENSEMBL Protists release 29 (37). The ANI values were converted to a matrix of distances, which in turn were used for principal-component analysis (PCA) and hierarchical clustering (R hclust function [https://www.r-project.org/]).

### Chromosome sequencing coverage.

For each read alignment file, Samtools view (version 1.3) and BEDTools genomecov (version 2.25.0) (38) were used to measure the sequencing depth of each nucleotide. Samtools was run with options “-q 50 -F 1028” to discard reads with a low map quality score or potential duplicates, while BEDTools genomecov was run with options “-d -split.” Nucleotide coverage was normalized by the median genomic coverage.

The chromosome sequencing coverage was used to evaluate aneuploidy between EP and EP + 3 samples. For each sample and for each chromosome, the median sequencing coverage was computed for contiguous windows of 2,500 bases. For those strains for which two EP + 3 samples were available, the mean of EP + 3.1 and EP + 3.2 was used to calculate the statistical significance of amplification compared to EP. The distributions of the median window coverage in EP and EP + 3 were compared by one-way analysis of variance (ANOVA). To have an estimate of the chromosome copy number differences, the window coverage was further normalized by chromosome 19 median coverage and multiplied by 2. For each chromosome, the median values in EP and EP + 3 were compared. Both the ANOVA *P* values and the chromosome somy comparisons are reported in Table S4 at GitLab (https://gitlab.pasteur.fr/gbussott/Leishmania_genome_dynamics_during_environmental_adaptation_reveals_strain_specific_differences/).

### Gene sequencing coverage.

Samtools view (version 1.3) and BEDTools coverage (version 2.25.0) were used to measure the mean sequencing depth of every annotated gene and were run, respectively, with options “-q 50 -F 1028” and “-d -split.” Possible intragenic gap regions were excluded from the calculation of the mean. Then the mean coverage of each gene was normalized by the median coverage of its chromosome. To account for GC content sequencing bias, the coverage values were corrected using a LOESS regression with a 5-fold cross validation to optimize the model span parameter. Genes supported by reads with a mean mapping quality (MAPQ) score of <50 were filtered.

To enable CNV analysis of gene arrays and genes sharing high sequence identity, we clustered the nucleotide sequences of the annotated genes into groups with cd-hit (version 4.6) (39). We used the length difference cutoff option “-s 0.9.” Then we realigned the clusters with MAFFT (40) and used T-Coffee seq_reformat (41) to select a representative gene per cluster (RefGene) showing the highest average sequence similarity to the other cluster members. If two genes had the same average similarity, then the shortest was chosen. We used bwa to build a database containing only the sequences of RefGene, adding ±50 bp of the 5′ and 3′ ends to ease the read alignment and the quantification of small RefGenes. We realigned EP samples against this database using bwa mem with the option “-M.” We then quantified the RefGene mean coverage (without considering the ±50-bp extension) with Samtools view and BEDTools coverage using the options “-F 1028” and “-d -split,” respectively. Values were normalized by the median coverage of the RefGene’s chromosome. Gene groups composed by members located on different chromosomes were negligible and discarded.

### Genome binning.

The reference genomes were divided into contiguous windows of a fixed length, and the sequencing coverage of each window was evaluated and compared across different samples. A window length of 300 bases was used for the shown scatter plots assessing genome-wide CNVs. Both the mean sequencing coverage normalized by the median chromosome coverage and the mean read MAPQ value were computed. To account for GC content sequencing bias, the coverage values were corrected using a LOESS regression with a 5-fold cross validation to optimize the model span parameter. The windows with a MAPQ score below 50 in either EP or EP + 3.1 were discarded. Poorly supported windows with a median or mean sequencing depth smaller than one-tenth of the median chromosome coverage both in EP and EP + 3.1 were also discarded. The windows with an EP + 3/EP coverage ratio outside the axis limits were placed on the edge (value of 3). In the genome browser tracks, the repeat elements and low-complexity regions were predicted with RepeatMasker (version 4.0.6) (RepeatModeler software; AFA Smit and R Hubley, RepeatModeler Open-1.0, 2008–2015 [http://www.repeatmasker.org]) using the options “-e crossmatch -gff -xsmall -s” in combination with Repbase (42) to identify Leishmania-specific and ancestral repeats.

A window length of 2,000 bases was used for the shown Circos plots assessing chromosome amplification. Mean sequencing coverage and mean MAPQ score of the reads aligning to that window were reported. The histogram function of Circos (version 0.68-1) (43) was used to visualize the coverage of the windows, using a cutoff of 3. Windows with mean MAPQ score below 50 or overlapping genomic gaps of over 1 kb were assigned a sequencing coverage of 1.

### Single-nucleotide variant analysis.

To call single nucleotide variants (SNVs), we used Freebayes (version v1.0.1-2-g0cb2697) (44) with options “–no-indels –no-mnps –no-complex –read-mismatch-limit 3 –read-snp-limit 3 –hwe-priors-off –binomial-obs-priors-off –allele-balance-priors-off –min-alternate-fraction 0.05 –min-base-quality 5 –min-mapping-quality 50 –min-alternate-count 2 –pooled-continuous.” The output was filtered to retain the positions with just one alternate allele with a minimum frequency of 0.9 and a minimum mean mapping quality of 20 for the reads supporting the reference or the alternative allele. SNVs mapping inside homopolymers (i.e., simple repeats of the same nucleotide) were filtered using a more stringent parameter, requiring at least 20 reads supporting the variant. The homopolymers were defined as the DNA region spanning ±5 bases from the SNV, with over 40% of identical nucleotides. We discarded SNVs with sequencing coverage above or below 4 median absolute deviations (MADs). The predicted SNVs are reported in Table S5 at GitLab (https://gitlab.pasteur.fr/gbussott/Leishmania_genome_dynamics_during_environmental_adaptation_reveals_strain_specific_differences/).

### Analysis of structural variants.

DELLY (version 0.6.7) (45) was run with option “-q 50” to predict balanced structural variations, including translocations and inversion. To reduce false predictions, the DELLY output was additionally filtered removing structural variants overlapping for more than 50% of their size with either assembly gaps or repetitive elements. Predictions mapping within 10 kb from the telomeric ends were removed to reduce false-positive results caused by possible misassembled regions close to the chromosome ends. Signals showing DELLY paired-end support of the structural variant (PE) or the high-quality variant pairs’ score (DV) inferior to 20 were removed, as well as signals showing high-quality variant pairs inferior to 20. The predicted structural variants were represented with Circos.

### Synteny analysis.

The synteny analysis was performed with SyntView (46), a software package originally designed to compare microbial genomes. The tool was adapted to browse interactively the genome of four Leishmania reference genomes and explore their syntenic relation: L. infantum JPCM5, L. donovani PBQ7IC8, L. major Friedlin, and L. donovani BPK282A1. This new tool hosting Leishmania syntenic data is publicly available at http://genopole.pasteur.fr/SynTView/flash/Leishmania/SynWebLinfantum.html.

### Supplementary table availability.

All supplemental tables are publicly available at GitLab at https://gitlab.pasteur.fr/gbussott/Leishmania_genome_dynamics_during_environmental_adaptation_reveals_strain_specific_differences/.

### Accession number(s).

Reads were deposited in the Sequence Read Archive database (SRA) database (47) and are publicly available under accession no. SRP126578.
