# Supplementary figures and images for: Leishmania Genome Dynamics during Environmental Adaptation Reveal Strain-Specific Differences in Gene Copy Number Variation, Karyotype Instability, and Telomeric Amplification
Source: mBio. 2018 Nov 6;9(6):e01399-18. doi: 10.1128/mBio.01399-18 (PMC6222132; doi:10.1128/mBio.01399-18)

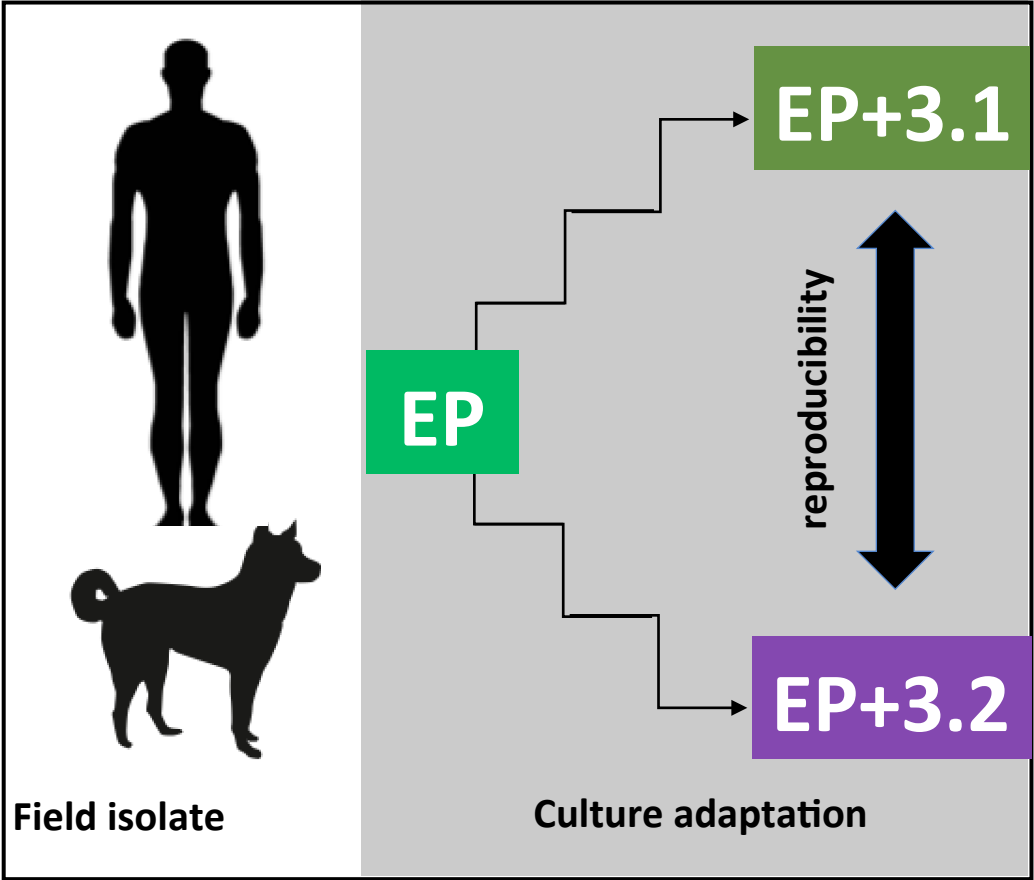

Supplement: FIG S1 [file mbo005184123sf1.pdf]

A

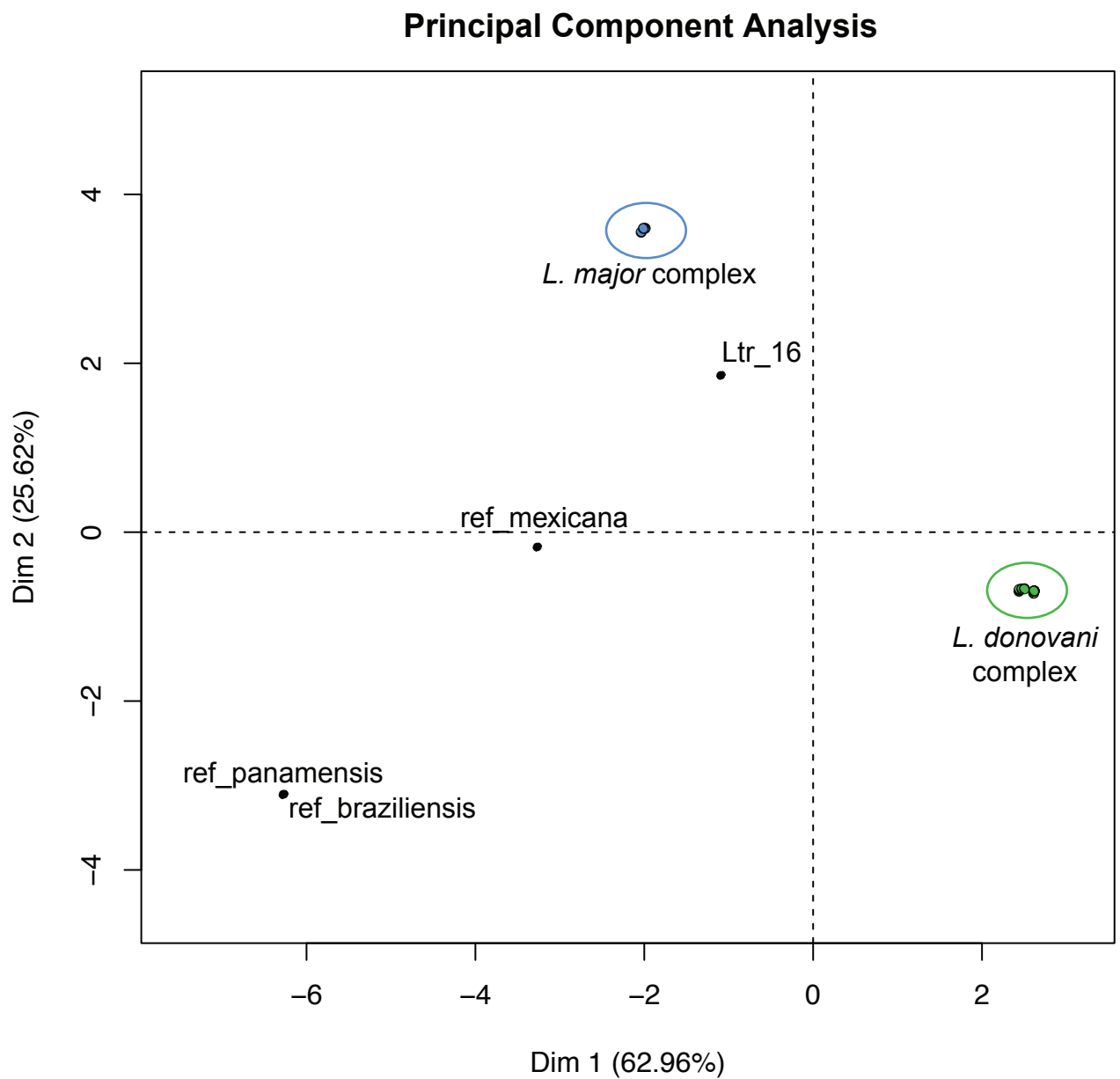

B

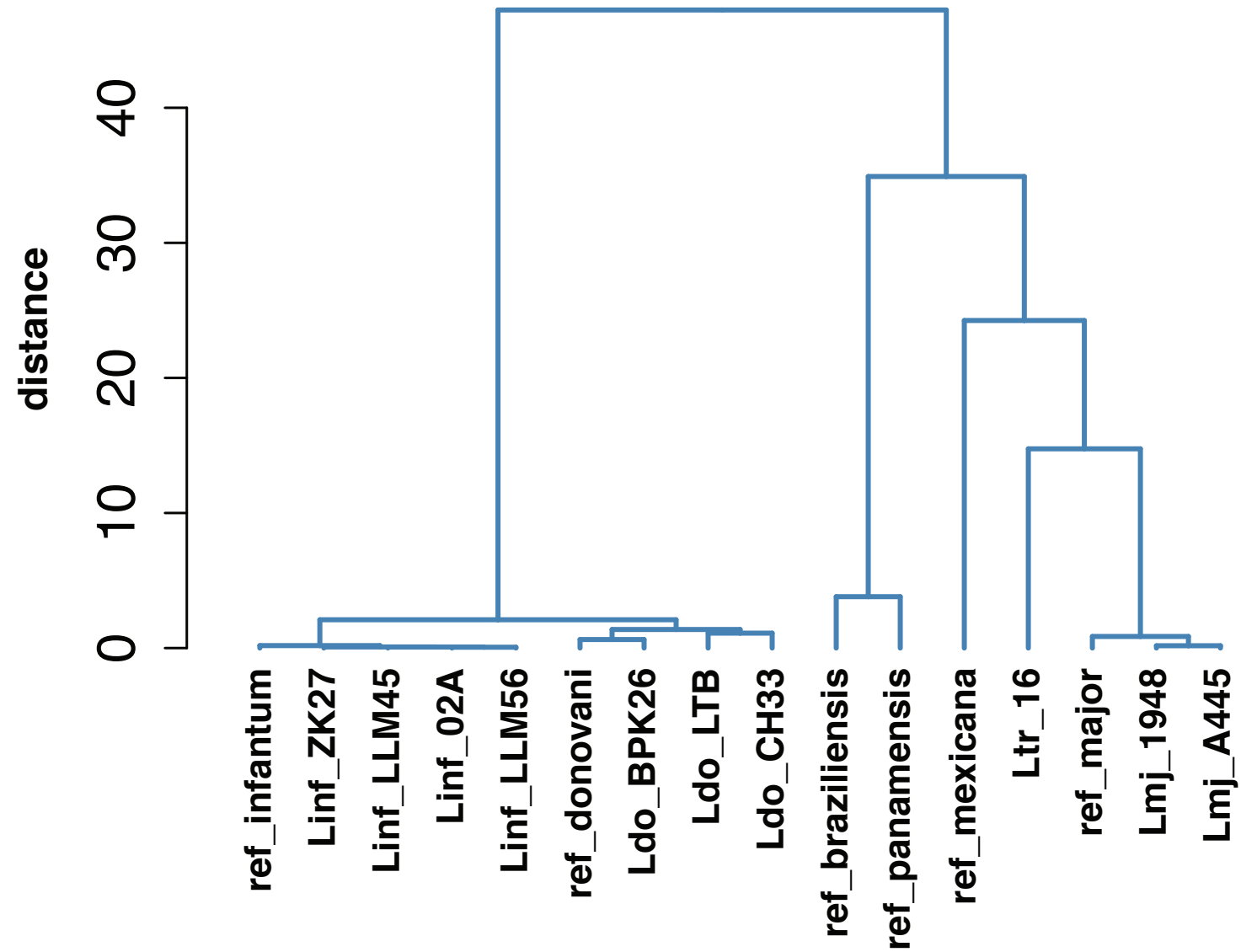

Supplement: FIG S2 [file mbo005184123sf2.pdf]

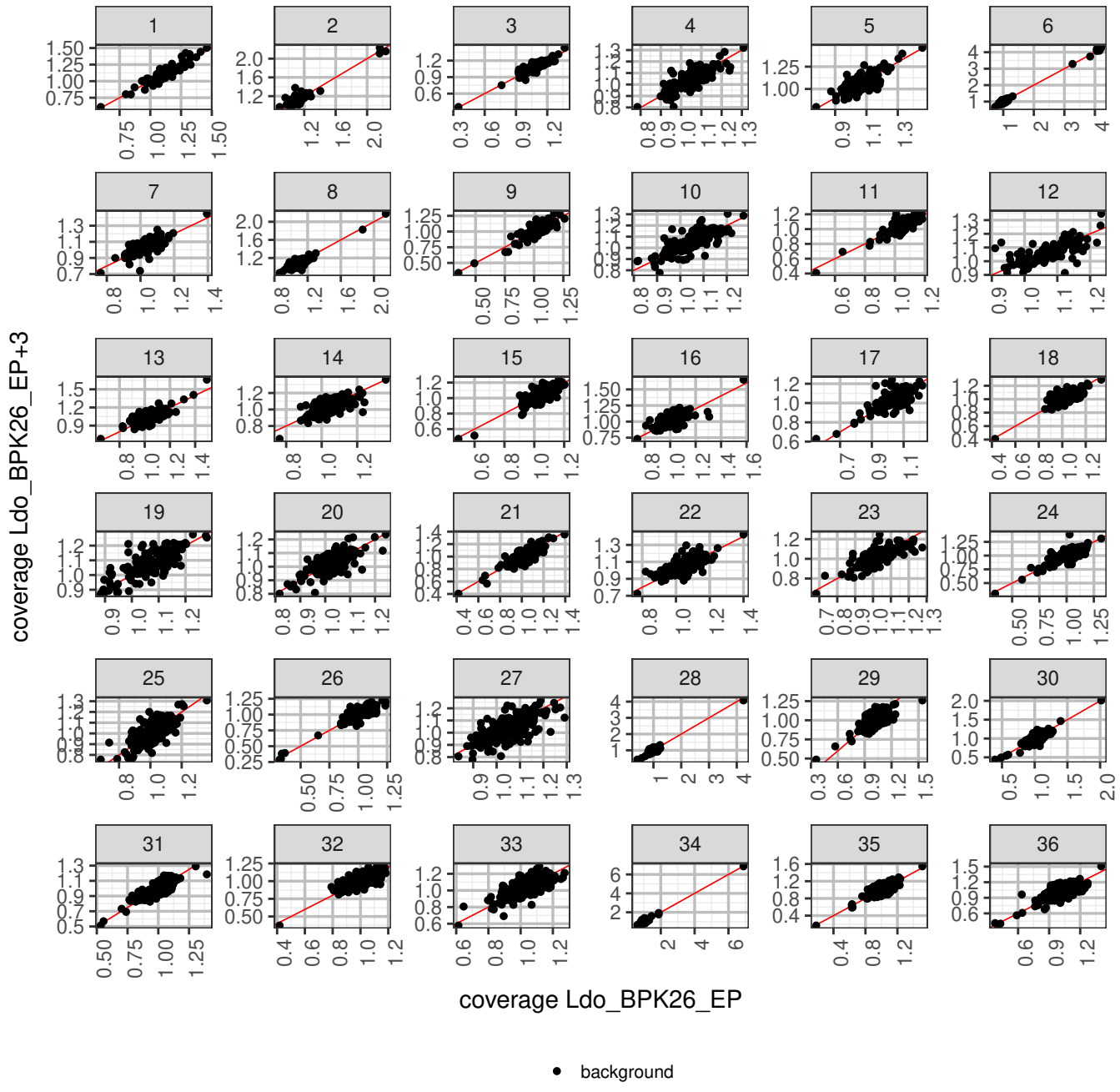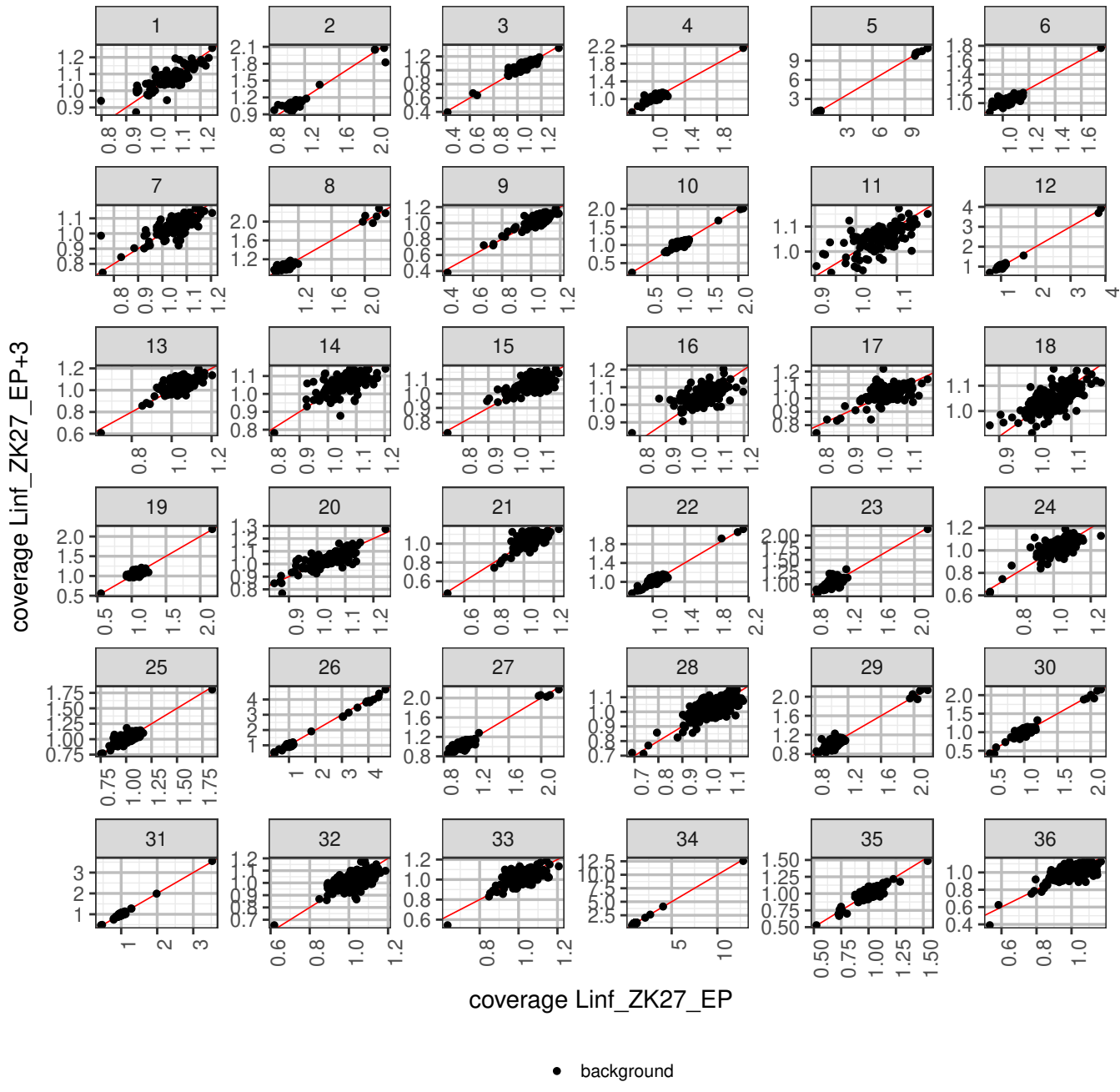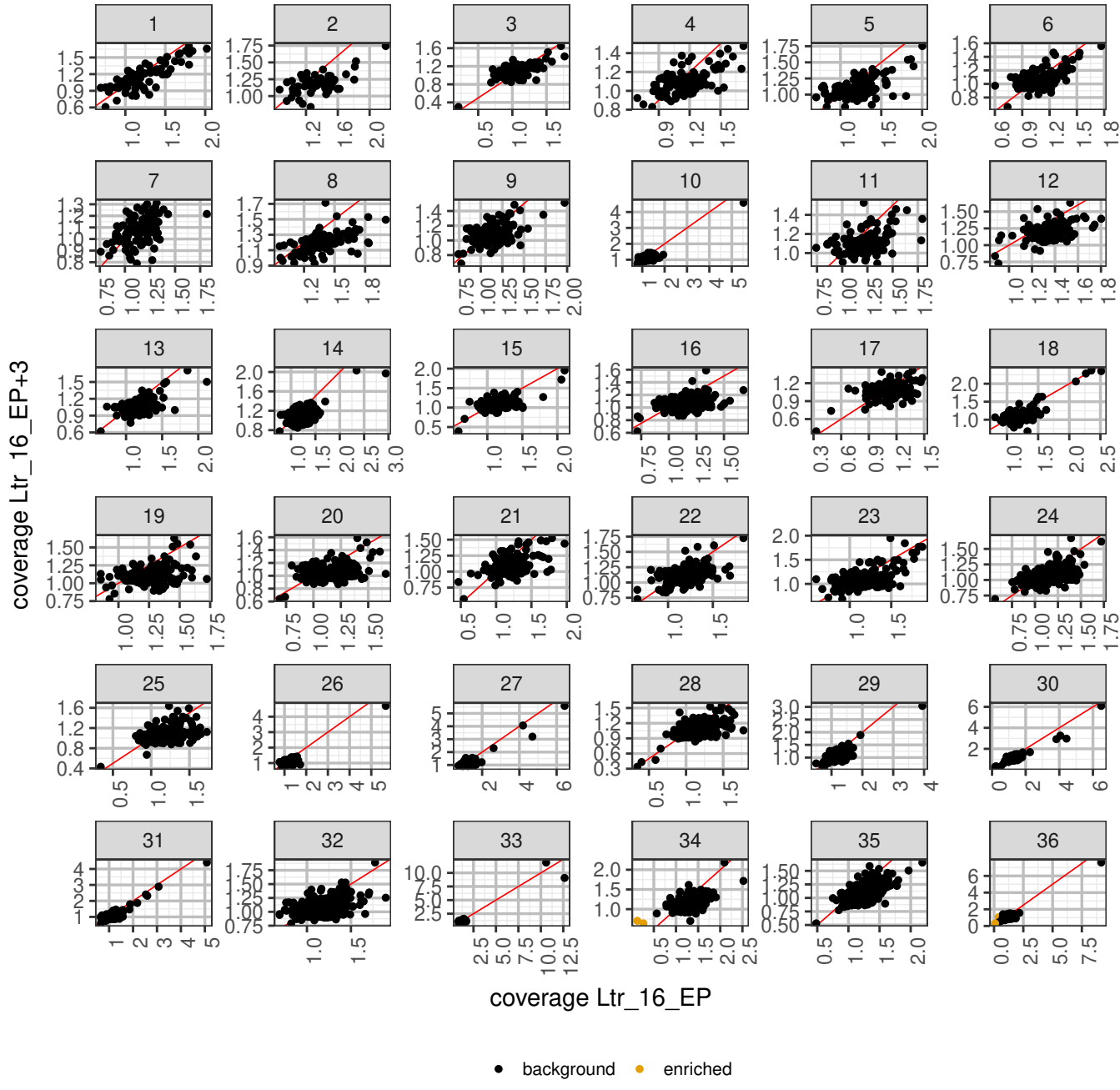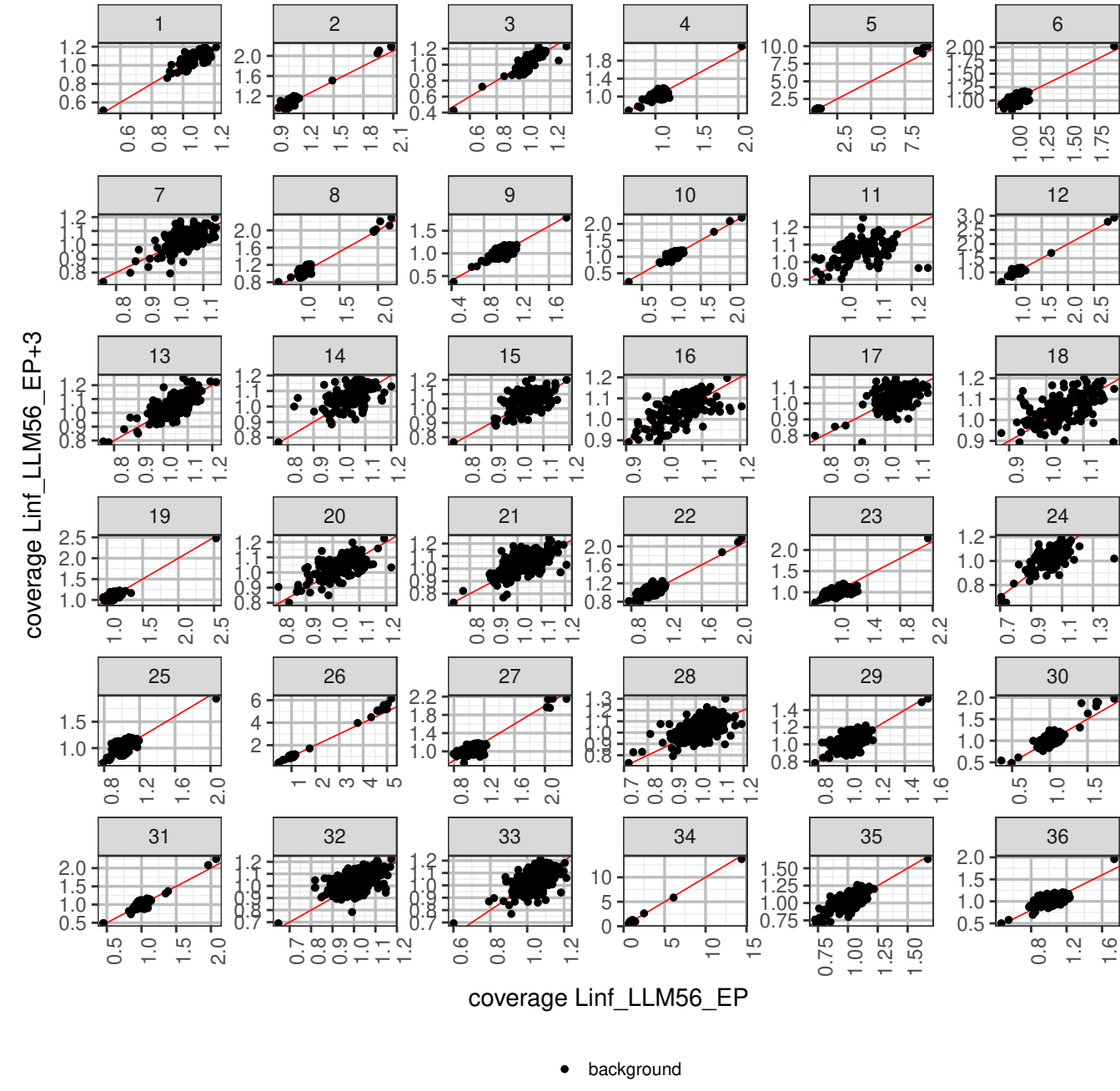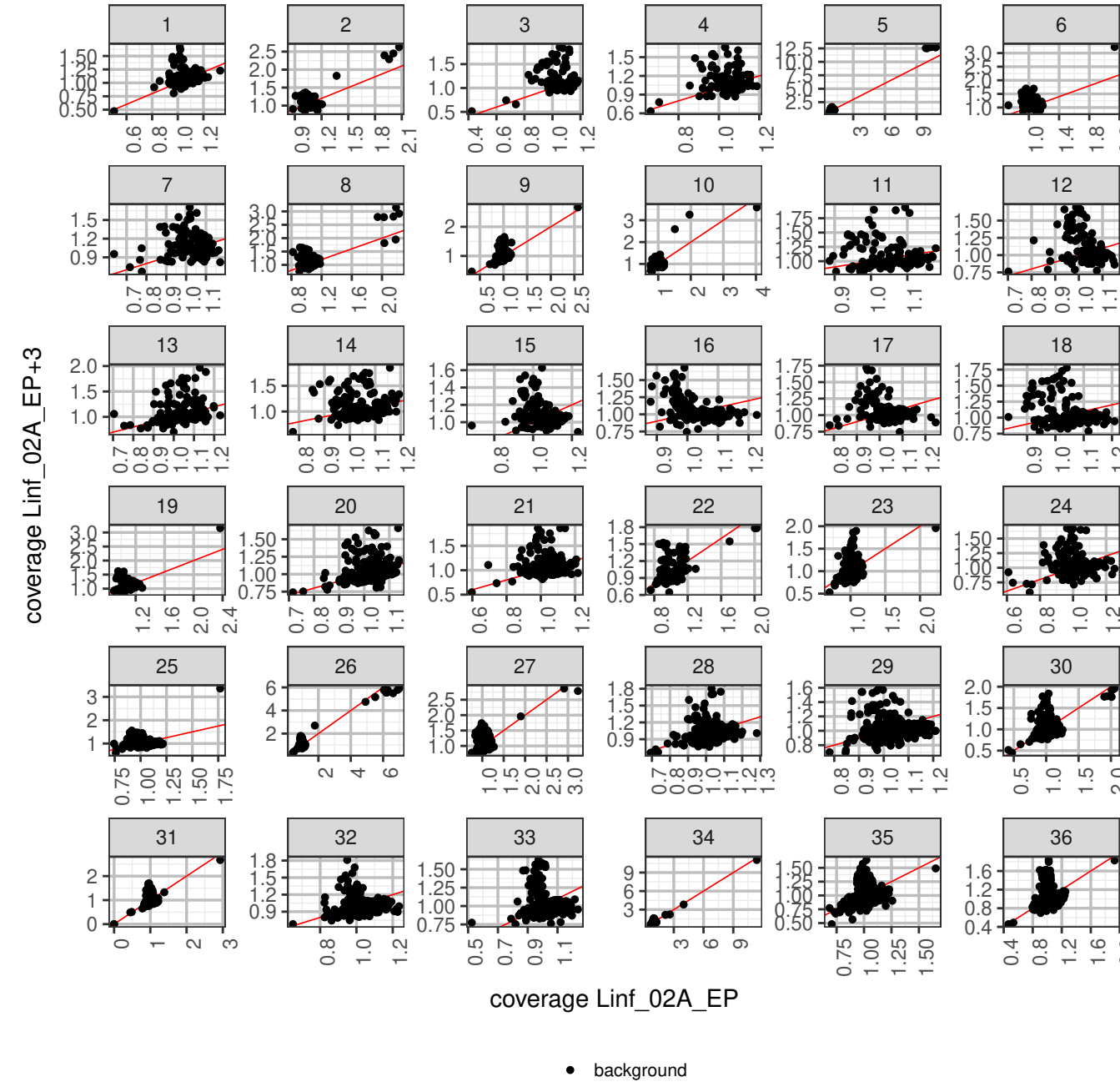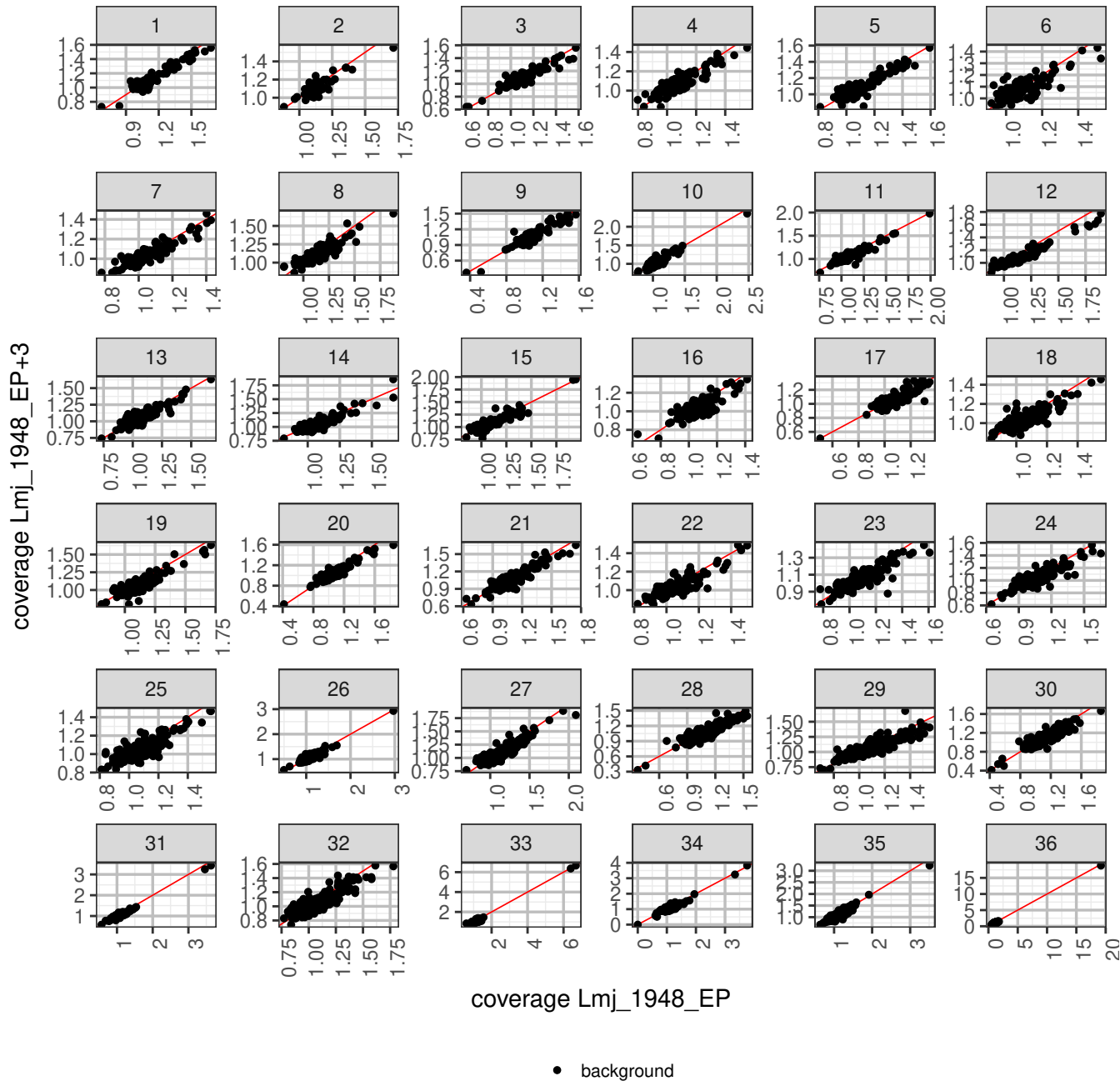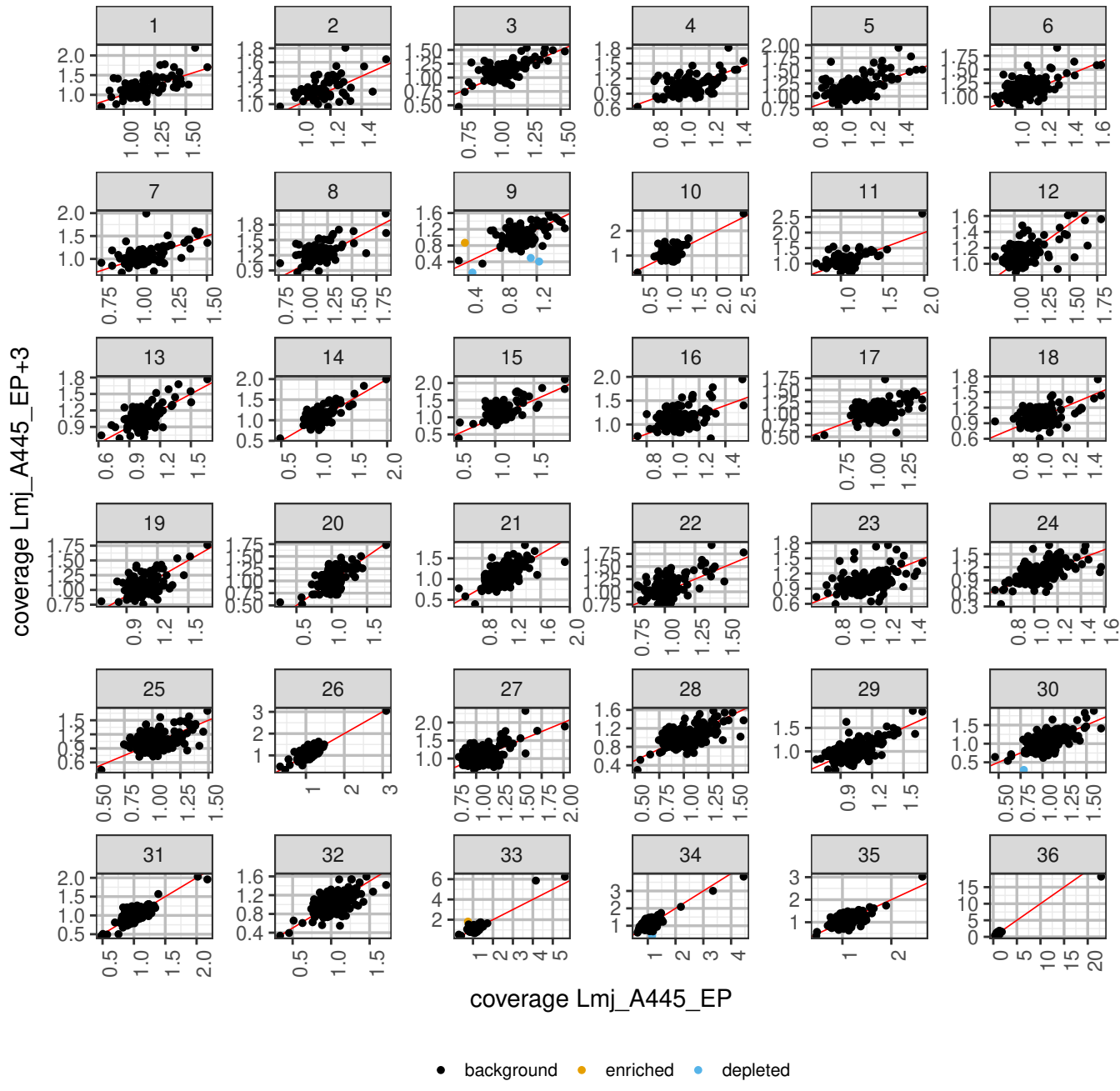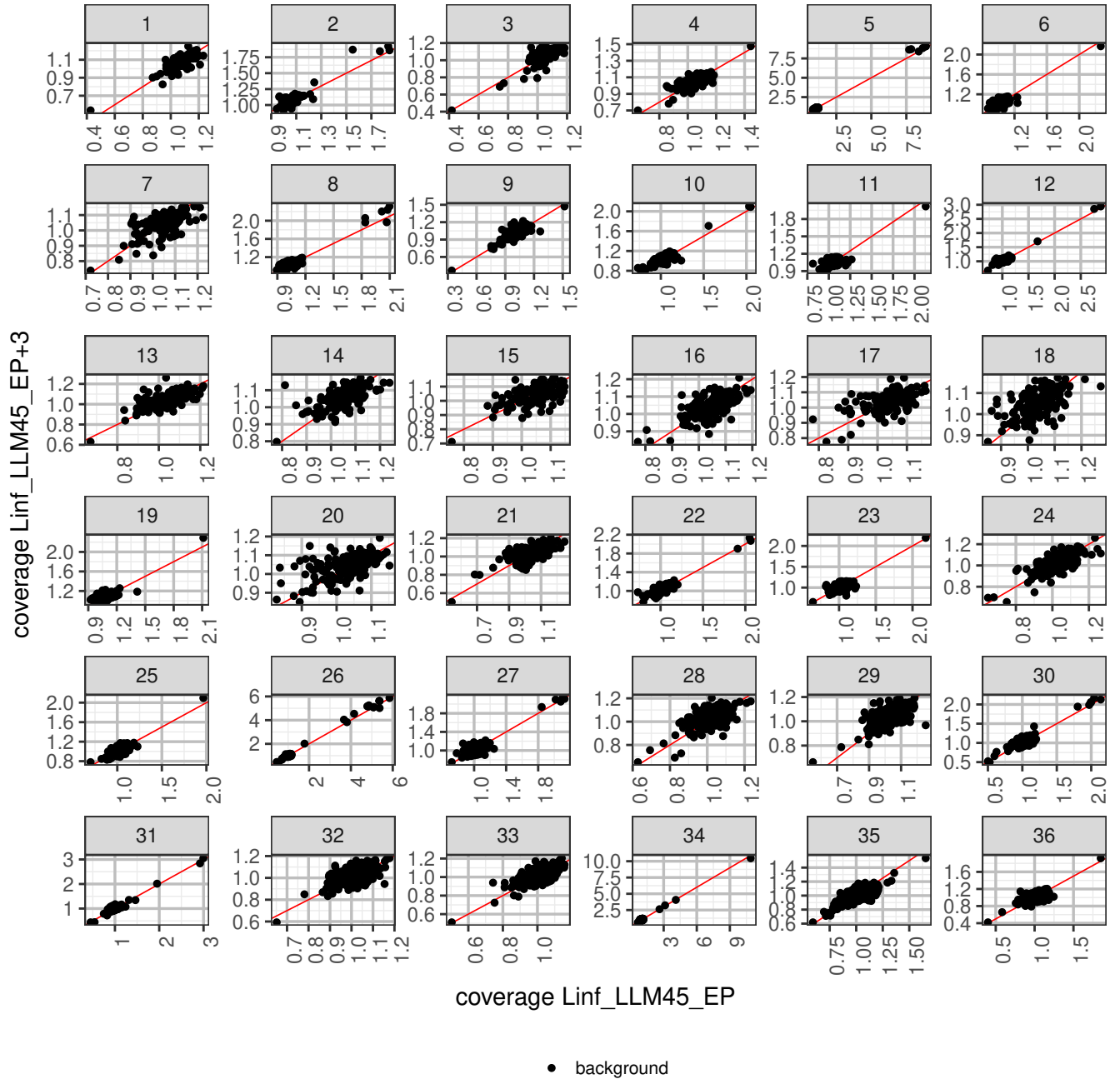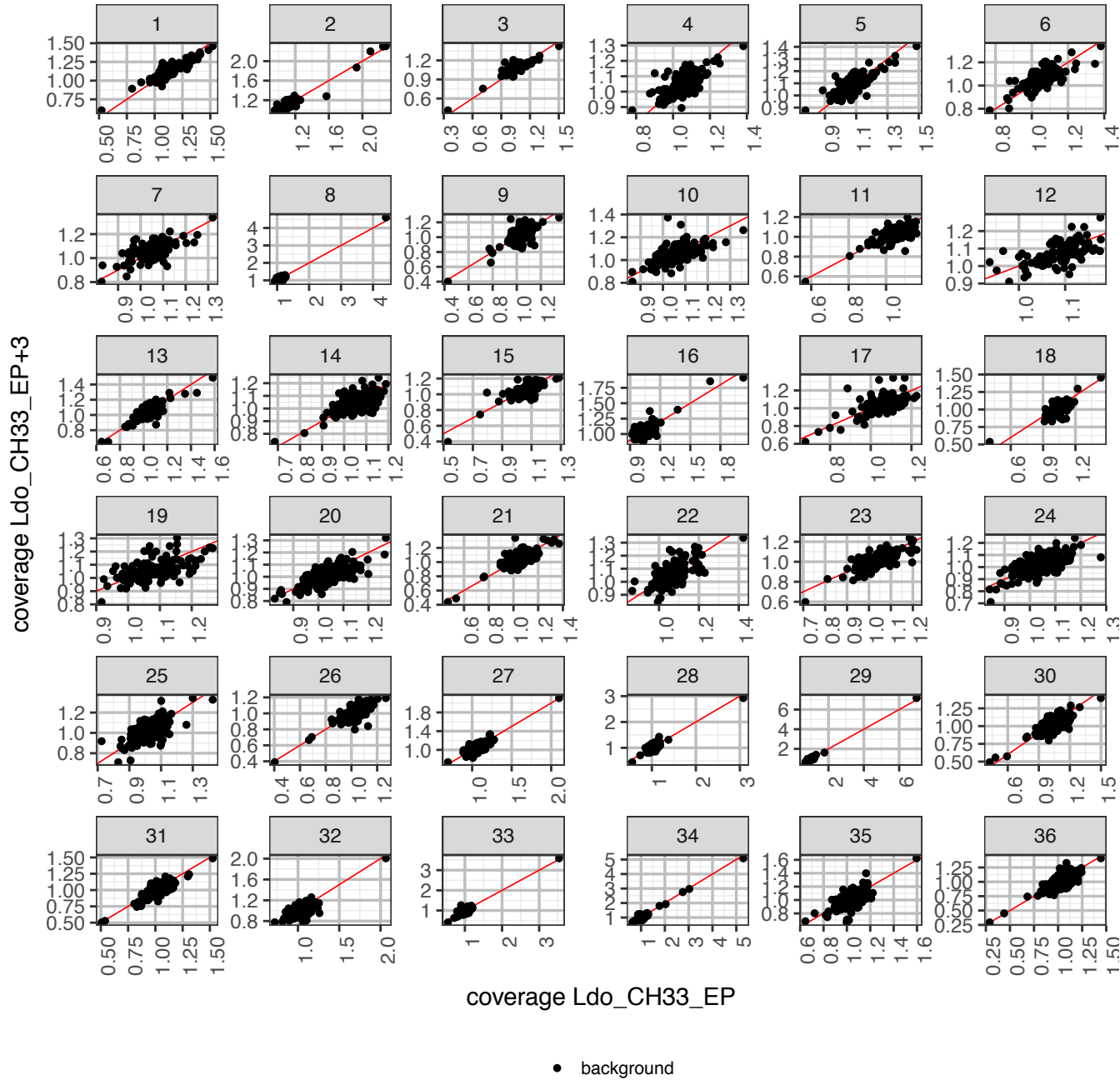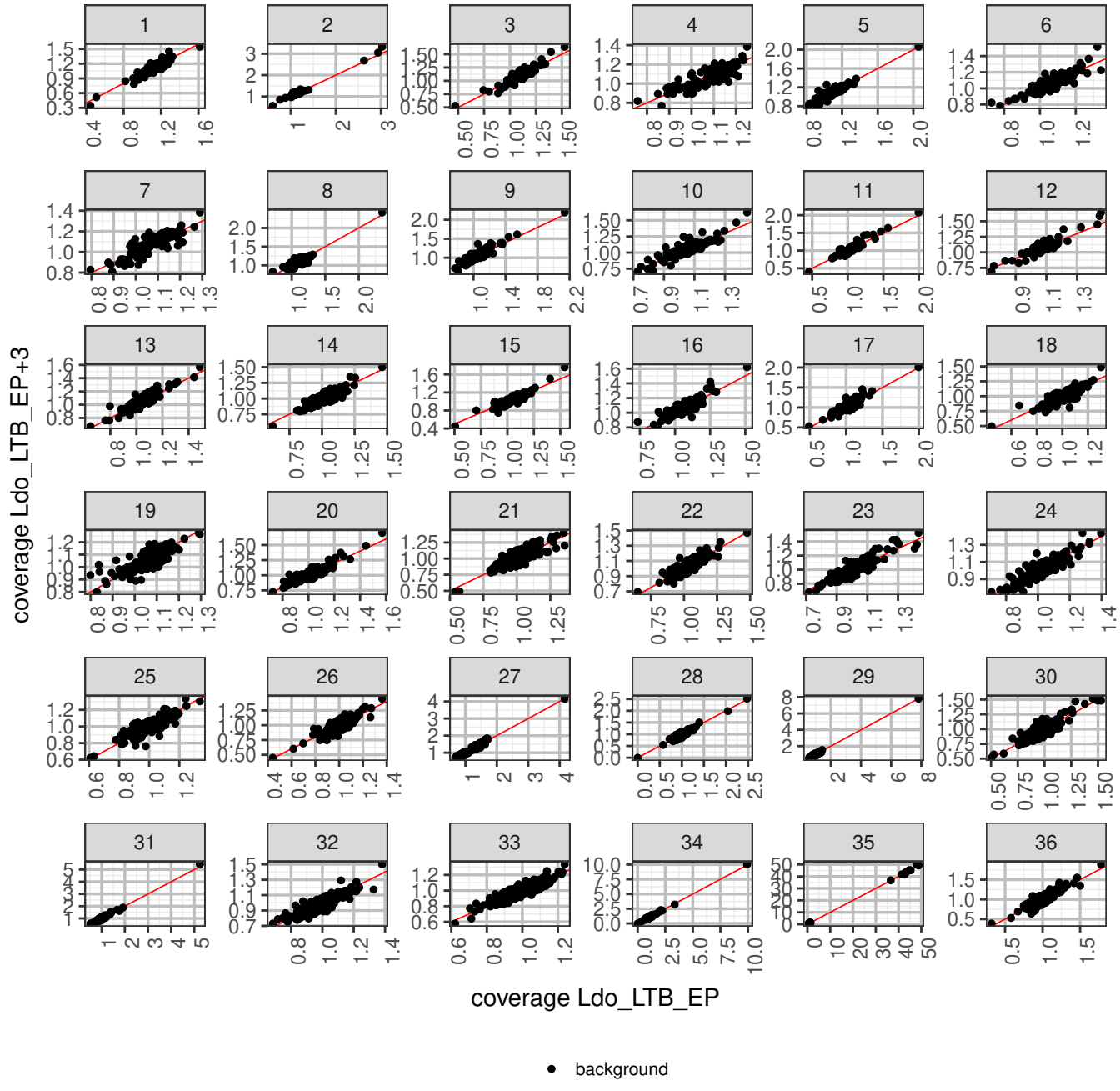

Supplement: FIG S4 [file mbo005184123sf4.pdf]
